# S-amlodipine induces liver inflammation and dysfunction through the alteration of intestinal microbiome in a rat model

**DOI:** 10.1080/19490976.2024.2316923

**Published:** 2024-02-24

**Authors:** Xinxin Liu, Hui Fang, Liuzhu Pan, Peng Zhang, Huai Lin, Huihui Gao, Chaolin Ye, Daqing Mao, Yi Luo

**Affiliations:** a College of Environmental Sciences and Engineering, Ministry of Education Key Laboratory of Pollution Processes and Environmental Criteria, Nankai University, Tianjin, China; b State Key Laboratory of Pollution Control and Resource Reuse, School of the Environment, Nanjing University, Nanjing, China; c School of Medicine, Nankai University, Tianjin, China

**Keywords:** S-amlodipine, liver dysfunction, gut microbiome, LPS, intestinal barrier damage, inflammatory response

## Abstract

S-amlodipine, a commonly prescribed antihypertensive agent, is widely used in clinical settings to treat hypertension. However, the potential adverse effects of long-term S-amlodipine treatment on the liver remain uncertain, given the cautionary recommendations from clinicians regarding its administration in individuals with impaired liver function. To address this, we conducted a study using an eight-week-old male rat model and administered a daily dose of 0.6 ~ 5 mg/kg of S-amlodipine for 7 weeks. Our findings demonstrated that 1.2 ~ 5 mg/kg of S-amlodipine treatment induced liver inflammation and associated dysfunction in rats, further *in vitro* experiments revealed that the observed liver inflammation and dysfunction were not attributable to direct effects of S-amlodipine on the liver. Metagenome sequencing analysis revealed that S-amlodipine treatment led to alterations in the gut microbiome of rats, with the bloom of *E. coli* (4.5 ~ 6.6-fold increase) and a decrease in *A. muciniphila* (1,613.4 ~ 2,000-fold decrease) and *B. uniformis* (20.6* ~ *202.7-fold decrease), subsequently causing an increase in the gut bacterial lipopolysaccharide (LPS) content (1.4 ~ 1.5-fold increase in feces). S-amlodipine treatment also induced damage to the intestinal barrier and increased intestinal permeability, as confirmed by elevated levels of fecal albumin; furthermore, the flux of gut bacterial LPS into the bloodstream through the portal vein resulted in an increase in serum LPS content (3.3 ~ 4-fold increase). LPS induces liver inflammation and subsequent dysfunction in rats by activating the TLR4 pathway. This study is the first to show that S-amlodipine induces liver inflammation and dysfunction by perturbing the rat gut microbiome. These results indicate the adverse effects of S-amlodipine on the liver and provide a rich understanding of the safety of long-term S-amlodipine administration.

## Introduction

Primary hypertension, a chronic disease, accounts for over 90% of all cases of hypertension and it is one of a risk factor for cardiovascular conditions.^1^ Hypertension has a global prevalence and is particularly prevalent in various countries and regions, including Central Asia, Oceania, Central and Eastern Europe, Southern Africa and Latin America.^2^ Hypertensive patients often require long-term medication to manage their conditions,^3^ with some individuals undergoing treatment for their entire lifespan; therefore, it is fairly important to evaluate antihypertensive drugs for medication safety. Currently, calcium channel blockers have been universally used in the treatment of hypertension due to their mild and reversible impact on liver function,^4,5^ even so, a few rare instances of symptomatic and severe liver damage have been occasionally reported after taking certain calcium channel blockers.^6,7^ S-amlodipine (S-enantiomer of amlodipine), an antihypertensive calcium channel blocker drug, inhibits L-type calcium channels and blocks the depolarization of cardiac myocytes and vascular smooth muscle cells.^8^ It has been widely prescribed for hypertension treatment in numerous countries, including China, America, Russia, Ukraine, Turkey, India, Japan, Korea, Mongolia, the Philippines, and Nepal.^9–12^ Generally, S-amlodipine undergoes a first-step hepatic metabolism after participating in the blood circulation system. Although the side effects of S-amlodipine on the liver have not yet been reported in the administrated population, clinicians recommend that hypertensive patients with impaired liver function use this drug with more caution. To date, it remains unclear whether long-term S-amlodipine use can cause adverse effects on the liver.

The human intestine harbors a complex and diverse commensal microbiota consisting of approximately 1,000 microbial species, which play vital roles in host physiology, lipid metabolism, energy harvesting and immune regulation.^13–16^ Previous studies have suggested that calcium channel blockers influence gut microbiota diversity.^17,18^ In general, drugs that interact with the gut microbiota can impact microbial metabolism.^19^ For example, amlodipine and nifedipine can be metabolized by the intestinal microbiota, which might change the composition of gut microbiota and influence drug absorption.^20,21^ However, the interaction between S-amlodipine and the gut microbiota remains unclear, especially considering its long-term administration as an antihypertensive drug.

In this study, we hypothesized that long-term oral administration of S-amlodipine may affect the gut microbiome and subsequently influence liver function. To test this hypothesis, we conducted fecal metagenome sequencing, analyzed immune indicators and alterations in the gut microbiome, and examined histomorphological changes in the liver and intestine using a rat model. To our knowledge, this is the first study to reveal the adverse effects of S-amlodipine. Considering the large population of hypertensive patients who are taking oral S-amlodipine chronically, this study shed a light for those patients to avoid the possible liver damage by rational drug use.

## Results

### Oral administration of S-amlodipine caused liver inflammation and associated dysfunction in rats

**Figure 1.**
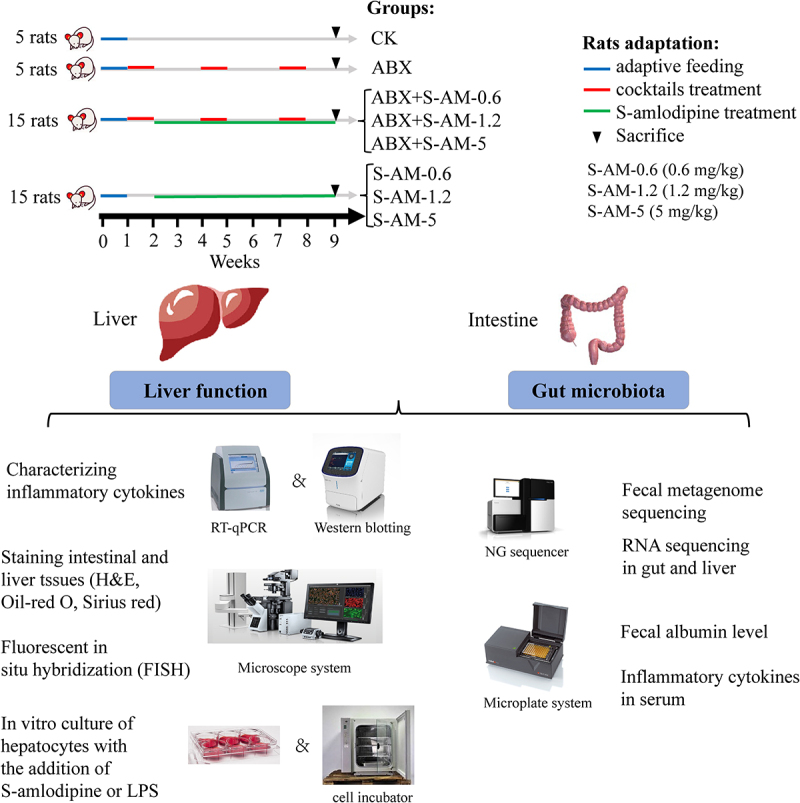
Experimental design and schematic summary in rats. Specific details of animal experimental design are shown in the materials and methods.

**Figure 2.**
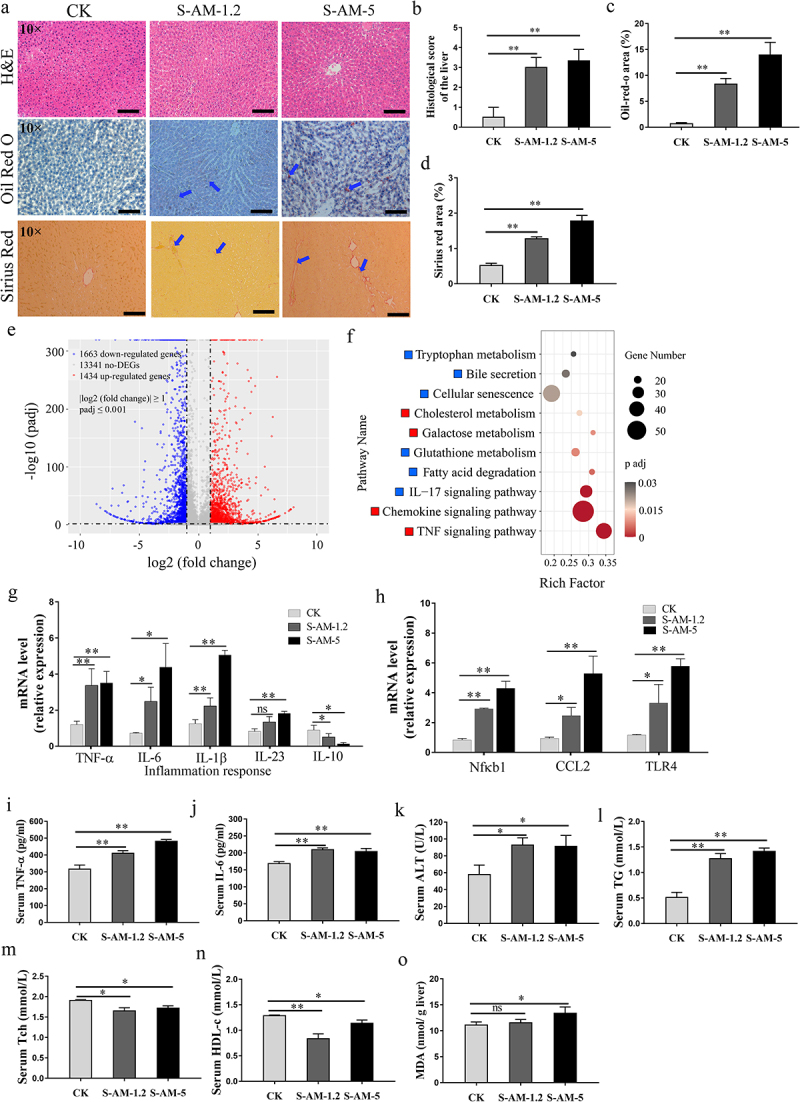
S-amlodipine induced hepatic inflammation and a shift in liver indexes in rats. (a) Representative pictures of liver sections after H&E, Oil Red O, or Sirius Red staining are shown in panels. Arrows indicate regions of lesions. (b-d) the histology score of the liver was evaluated (b), and the quantitation of the oil-red area (c) or the fibrosis area (d) was measured. Scale bars, 50 μm. *N* = 3 per group. (e) Volcano plot showing the comparative transcriptomic analysis (visualization of DEGs) of the liver in the S-AM-5 group versus the CK group. (f) KEGG enrichment analysis of DEGs in the S-AM-5 group versus the CK group based on RNA-seq. The red squares represent significantly upregulated pathways, while blue squares represent significantly downregulated pathways. (g) The mRNA expression of inflammatory genes in the liver were detected by RT-qPCR. *N* = 5 per group. (h) The mRNA expression of Nfκb1, chemokine CCL2 and TLR4 genes in the liver were detected by RT-qPCR. *N* = 5 per group. (i-j) serum level of TNF-α (i), and IL-6 (j) were measured by ELISA. *N* = 5 per group. (k-n) the serum ALT (k), TG (l), Tch (m), and HDL-c (n) level in rats. *N* = 3 per group. (o) Hepatic MDA level. *N* = 5 per group. ALT, alanine transaminase; TG, triglycerides; Tch, total cholesterol; HDL-c, high-density lipoprotein cholesterol. MDA, malondialdehyde. Significance was determined using t test analysis. *p < 0.05, **p < 0.01.

To evaluate the effects of S-amlodipine on the liver, we treated rats with S-amlodipine administered by oral gavage at different doses (0.6, 1.2, or 5 mg/kg) once daily for 7 weeks (Figure 1). We observed that treatment with 0.6 mg/kg of S-amlodipine did not cause liver inflammation or associated dysfunction in rats (Figure S1). Although treatment with 1.2 ~ 5 mg/kg of S-amlodipine resulted in a decrease in the body weight of the rats after day 14 (Figure S2a) and there was no significant change in the ratio of liver weight to body weight (Figure S2(b-c)), increased hepatocyte ballooning (Hematoxylin and Eosin (H&E) staining), fat deposition (Oil Red O staining) and liver fibrosis areas (Sirius Red staining), indicating partial hepatocyte injury (Figure 2(a–d)). Once hepatocytes undergo damage, they release specific molecules that can activate the regulatory mechanisms of the hepatic immune cells, which may cause liver inflammation. To explore the inflammatory response in S-amlodipine-treated rats, a hepatic transcriptome sequencing analysis was conducted to reveal the overall landscape of mRNA expression. In this study, we observed that 5 mg/kg of S-amlodipine treatment upregulated 1,434 genes and downregulated 1,663 genes (Figure 2(e)). Among the differentially expressed genes (DEGs), several were involved in liver immunity and inflammation, including *TNF-αip3*, *TNF-αip8*, *TNF-αip2*, *TNF-αip8l2*, *IL-1β*, *IL-18*, *IL-31ra*, *IL-1α*, *IL-2 rγ*, and *IL-10 rα* (Supplementary file 2). In addition, the Kyoto Encyclopedia of Genes and Genomes (KEGG) analysis showed that pathways associated with liver inflammatory responses, such as the TNF signaling pathway, chemokine signaling pathway, and cholesterol metabolism pathway, were upregulated in S-amlodipine-treated rats (Figure 2(f) and Supplementary file 3). In contrast, glutathione (GSH) metabolism pathway and bile secretion pathway were downregulated. Subsequently, we examined the expression of the downstream gene markers of liver inflammation associated with these pathways. We found remarkably upregulated expression of the marker genes of inflammatory indicators (*TNF-α*, *IL-6*, *IL-1β*, *IL-23*, and *Nfκb1*), chemokine *CCL2*, and toll-like receptor 4 (*TLR4*, an important pathogen recognition receptor that recognizes mainly LPS of Gram-negative bacteria) in S-amlodipine-treated rats compared with control rats (Figure 2(g–h)). Moreover, we found that treatment with S-amlodipine dramatically enhanced the levels of serum inflammatory indicators (tumor necrosis factor alpha (TNF-α) and IL-6) (Figure 2(i,j)), which might cause low-grade inflammation in rats. Collectively, these results suggest that the 7-week treatment with 1.2 ~ 5 mg/kg of S-amlodipine triggered liver inflammation in rats.

Previous studies have highlighted that uncontrolled progression of liver inflammation can cause changes in liver function.^22,23^ We subsequently examined markers of liver function and observed that rats treated with 1.2 ~ 5 mg/kg of S-amlodipine exhibited elevated serum levels of alanine transaminase (ALT) and triglyceride (TG) (Figure 2(k,l)), which could result in liver dysfunction. Moreover, the serum levels of total cholesterol (Tch) and high–density lipoprotein cholesterol (HDL-c) were decreased in S-amlodipine-treated rats, indicating that S-amlodipine treatment caused a cholesterol metabolism disorder (Figure 2(m,n)). Compared with control rats, those treated with 1.2 mg/kg of S-amlodipine showed no significant change in the level of hepatic MDA (Figure 2(o)). However, 5 mg/kg of S-amlodipine treatment increased the level of hepatic MDA (Figure 2(o)), decreased the oxidative stress response by increasing hepatic ROS, lowered hepatic GSH levels, stimulated the expression of the oxidative stress response gene *NOX2*, and reduced the expression of *Pparg* in liver tissues (Figure S3), resulting in liver redox dysbiosis in the S-amlodipine-treated rats.

To investigate whether the observed liver inflammation caused by S-amlodipine was a result of direct effects on the liver, we performed an *in vitro* experiment in which the reference concentration (1.25 ~ 20 μm) of S-amlodipine in the human liver and placebo (0 μm S-amlodipine) were chosen to treat HepG2 cells, and the placebo-treated cells were used as a control. We observed that the cell viability, as well as indicators of liver inflammation and oxidative stress, including hepatocellular malonaldehyde (MDA) level, markers of inflammatory indicator (TNF-α) level, oxidative stress indicator (GSH) level, and lipid droplet accumulation (Oil-Red-O area), were not significantly changed by 1.25 ~ 20 μm of S-amlodipine-treated hepatocytes compared with the placebo-treated control (Figure S4(a-f)). This demonstrated that S-amlodipine-induced liver inflammation and associated dysfunction in rats were not due to direct effects on the liver.

### The alterations in the gut microbiome of rats under S-amlodipine treatment

**Figure 3.**
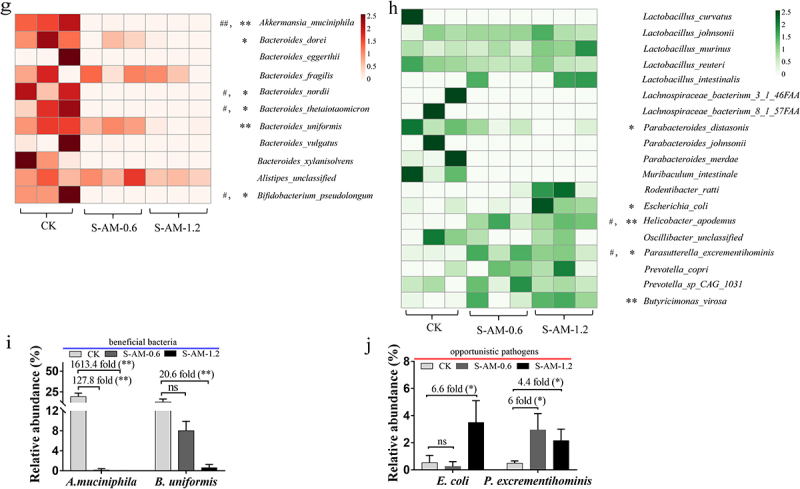
(Continue).

S-amlodipine administered to rats by oral gavage is primarily absorbed through the gastrointestinal tract. Whether long-term oral administration of S-amlodipine causes disturbances in intestinal microbiota remains unknown. In this study, we observed that oral gavage with S-amlodipine for seven weeks significantly perturbed the gut microbiota composition in rats. As shown in Figures 3(a,c,f) and S5(a,c,f), principal component analysis (PCA) indicated a distinct clustering of microbial taxa between the S-amlodipine-treated group and the control group, implying a substantial difference in the structure of the gut microbiota following S-amlodipine treatment. Specifically, phylum-based analysis revealed that the relative abundance of *Bacteroidetes* and *Verrucomicrobia* decreased, whereas *Firmicutes* and *Proteobacteria* increased after 1.2 ~ 5 mg/kg of S-amlodipine treatment (Figures 3(b) and S5(b)). At the genus level, 1.2 ~ 5 mg/kg of S-amlodipine treatment led to a substantial increase in the relative abundance of *Lactobacillus*, *Eschericha*, *Helicobacter*, *Butyricimonas*, *Pseudomonas*, *Oscillibacter* and *Parasutterella*, while reducing the abundance of *Akkermansia*, *Bacteroides*, *Alistipes*, and *Bifidobacterium* (Figures 3(d,e) and S5(d,e)). To reveal the changes at the species level after S-amlodipine treatment, metagenome sequencing analysis was performed on fecal samples. We observed a significant decrease in the relative abundance of beneficial bacteria; for example, *A. muciniphila*, *B. uniformis*, *A. shahii*, *B. thetaiotaomicron*, *B. nordii*, *B. dorei*, *B. pseudolongum* and *A. senegalensis* was decreased by 19.41 ~ 29.58%, 12.3 ~ 13.93%, 7.15%, 0.72 ~ 0.76%, 0.22 ~ 0.23%, 0.23 ~ 2.79%, 0.79 ~ 0.86% and 0.09%, respectively (Figures 3(g) and S5(g)), with the most significant decrease for *A. muciniphila* (1,613.4 ~ 2,000-fold decrease) and *B. uniformis* (20.6 ~ 202.7-fold decrease) (Figures 3(i) and S5(i)). It has been previously reported that these beneficial bacteria (*A. muciniphila*, *B. uniformis, B. thetaiotaomicron*, *B. nordii*, *B. dorei* and *B. pseudolongum*) have been associated with alleviating gastrointestinal immunological dysfunction and metabolic disorders,^24–26^ *A. muciniphila* has been reported to regulate intestinal barrier function by residing in the intestinal mucus layer.^27^ A decrease in *A. shahii* in the intestine has also been observed to be closely associated with inflammation in individuals with colorectal cancer or liver cirrhosis.^28^ In addition, the relative abundance of opportunistic pathogens such as *E. coli* and *P. excrementihominis* was greatly enriched in the S-amlodipine group (S-AM-1.2 and S-AM-5) (Figures 3(h) and S5(h)), with a 4.5 ~ 6.6-fold increase in *E. coli* and a 4.4 ~ 317.1-fold increase in *P. excrementihominis* (Figures 3j and S5(j)). Previous studies have shown that *E. coli* can lead to Crohn’s disease, diarrhea, sepsis, and urinary tract infection,^29,30^ and enriched *P. excrementihominis* has been associated with intestinal and liver diseases, such as inflammatory bowel disease, irritable bowel syndrome, and fatty liver disease.^31^ Moreover, *H. apodemus* was notably enriched in the S-AM-1.2 group, which might be associated with intestinal inflammation.^32^ Collectively, 1.2 ~ 5 mg/kg of S-amlodipine treatment diminished the relative abundance of beneficial bacteria and increased the relative abundance of opportunistic pathogens, causing significant changes in the intestinal microbiota composition of rats.
**Figure 3.**
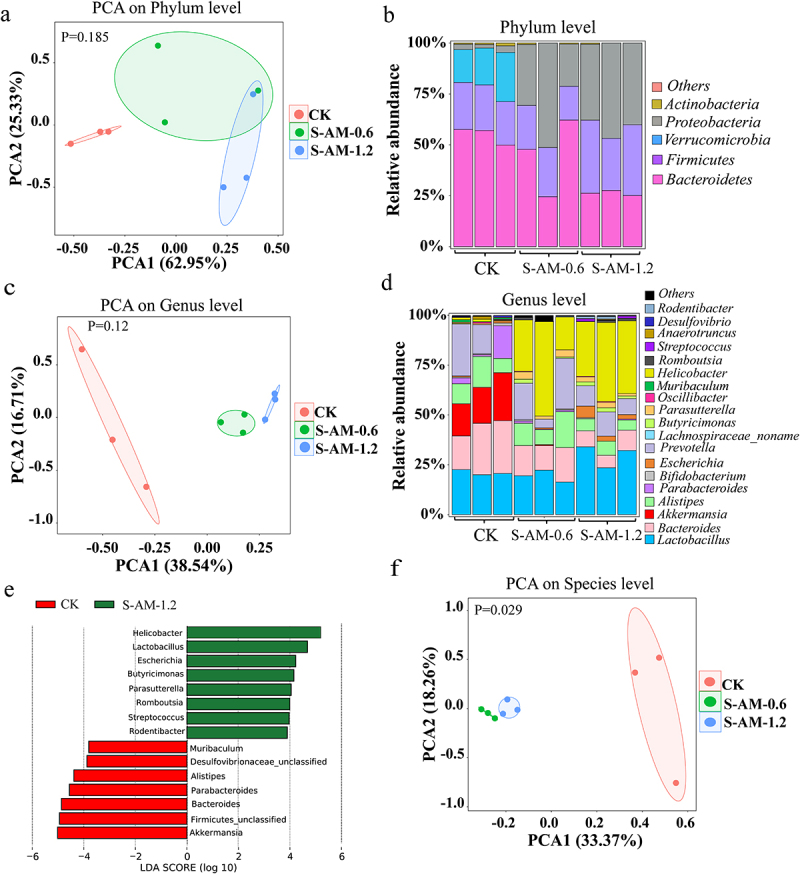
Metagenomic sequencing analysis uncovers obvious alterations in the composition and structure of the gut microbiome following 0.6 ~ 1.2 mg/kg of S-amlodipine treatment. (a), (c) and (f) Principal component analysis (PCA) of nine samples (CK group (*N* = 3) and S-amlodipine group (S-AM-0.6 and S-AM-1.2, *N* = 3 per group) based on phylum (a), genus (c) and species (f) level. (b) and (d) relative abundance of fecal bacteria at the phylum level (b) and genus level (d). (e) Relative abundance of bacteria in the gut was further analyzed using LEfSe analysis. (g) and (h) Heatmaps generated using R studio shows the taxonomic abundance at the species level based on metagenomic sequencing analysis results. The data is standardized. *N* = 3 per group. ^#^, *p* < 0.05, and ^##^, *p* < 0.01, S-AM-0.6 group compared with the CK group; *, *p* < 0.05, and **, *p* < 0.01, S-AM-1.2 group compared with the CK group. (i-j) comparison of the taxonomic abundance of main species potentially associated with the production of LPS was performed based on metagenomic sequencing analysis. Significance was determined using t test analysis. Data are presented as the mean ± SEM. *p < 0.05, **p < 0.01.

### S-amlodipine-induced the alteration of gut microbiome caused the intestinal inflammation and the increasement of intestinal permeability

**Figure 4.**
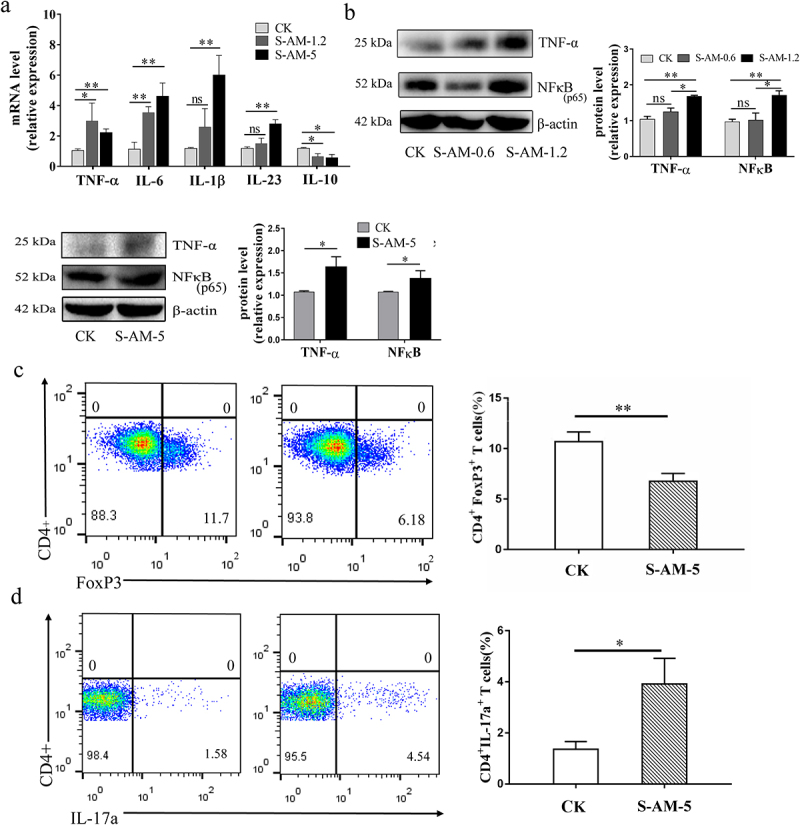
S-amlodipine treatment induces intestinal inflammation by altering the gut microbiome. (a) The mRNA expression of inflammatory genes in the colon was detected by RT-qPCR. *N* = 5 per group. (b) Representative images of western blot analysis on colon tissues were obtained, and the protein expression of TNF-α and NFκB was quantified. (c-d) FACS analysis was performed on colon-isolated immune cells (Treg cells (c) and Th17 cells (d)), and the data was quantified. *N* = 3 per group. Values are mean ± SEM. *p < 0.05, **p < 0.01.

Intestinal dysbiosis caused by detrimental fluctuations in the gut microbiome has been shown to be associated with the development of intestinal inflammation.^33^ In our study, we found that 1.2 ~ 5 mg/kg of S-amlodipine triggered intestinal inflammation in rats, which might be associated with the enrichment of opportunistic pathogens such as *E. coli* and *P. excrementihomini*s. Previous studies have demonstrated that the increased abundance of *E. coli* and *P. excrementihomini*s in the intestine is related to chronic intestinal inflammation in mice.^34,35^ Additionally, a study showed that Enteroaggregative *E. coli* isolated from healthy volunteers can induce an inflammatory response in human intestinal epithelial cells *in* *vitro*.^36^ In this study, we also investigated the markers of inflammatory indicators and found that the expression of proinflammatory cytokine genes (*TNF-α*, *IL-6*, *IL-1β* and *IL-23*) and proteins (TNF-α and NF-κB) was notably upregulated in the intestine after 1.2 ~ 5 mg/kg of S-amlodipine treatment (Figure 4(a,b)). Conversely, mRNA expression of the anti-inflammatory gene *IL-10* was downregulated. In addition, flow cytometry analysis results showed a decrease in colon-isolated CD4^+^ FoxP3^+^ T cells and an increase in CD4^+^ IL-17a^+^ T cells in 7-week S-amlodipine-treated rats compared with control rats (Figure 4(c,d)). However, treatment with 0.6 mg/kg of S-amlodipine did not induce intestinal inflammation in rats (Figure S6(a)). Taken together, these results demonstrate that 1.2 ~ 5 mg/kg of S-amlodipine triggers intestinal inflammation by altering the gut microbiome, which is characterized by elevated *E. coli*, boosting the upregulation of proinflammatory factors and the regulatory response of immune cells.

**Figure 5.**
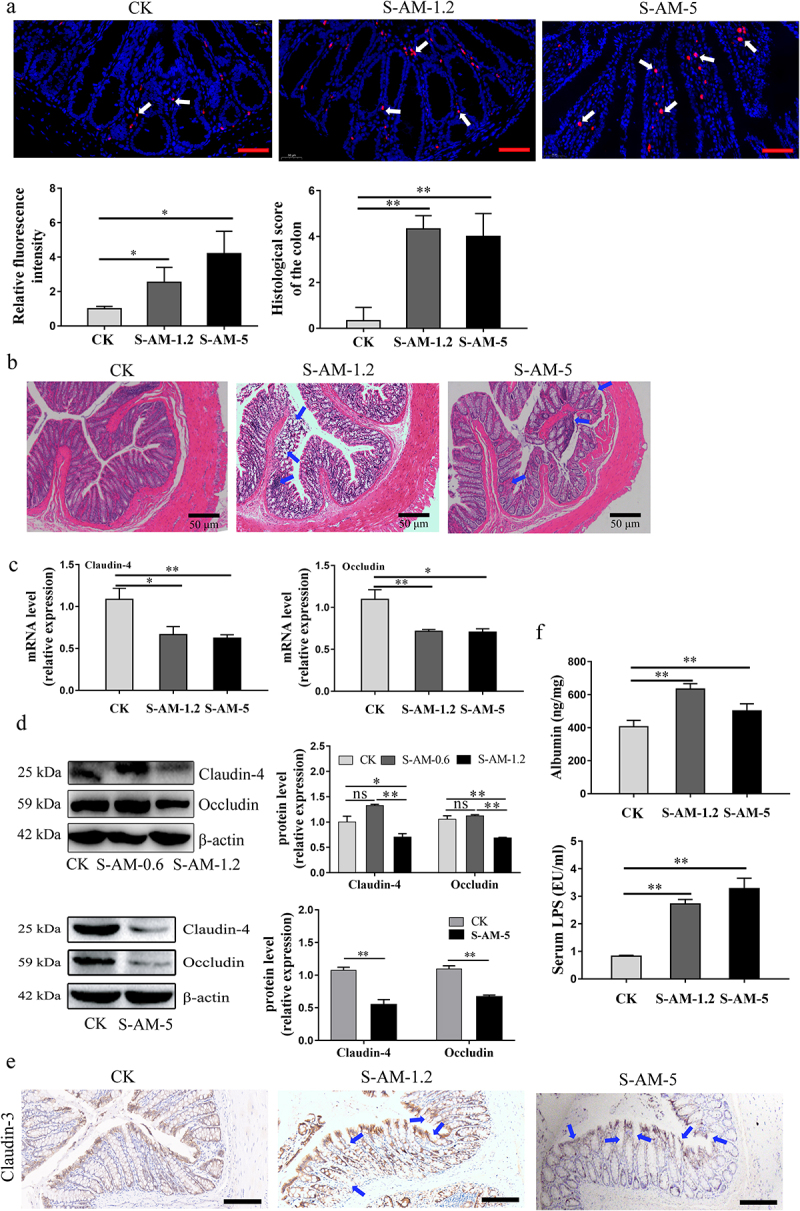
The alteration of the gut microbiome induced by S-amlodipine damages the intestinal barrier function and motivates bacterial invasion into the mucus layer in rats. (a) Bacterial 16s rRNA was stained using the EUB338 probe in colon sections and the relative fluorescence intensity (FI) was calculated utilizing ImageJ software. *N* = 3 per group. Scale bars, 25 μm. (b) Representative images of HE staining of colons were obtained, and the histology score of the colons was determined. *N* = 3 per group. (c) RT-qPCR analysis on colon extracts was performed to assess the mRNA expression of claudin-4 and occludin, which showed a decline in the S-amlodipine group. *N* = 5 per group. (d) Representative images of western blot analysis on colon tissues were obtained, and the protein expression of claudin-4 and occludin was quantified. (e) Representative images of immunochemistry staining for distribution of claudin-3 in colon tissues. *N* = 3 per group. Scale bars, 50 μm. (f) The fecal albumin contents and the serum LPS level in rats were measured. *N* = 5 per group. *p < 0.05, **p < 0.01. Arrows indicate regions of lesions.

Chronic inflammatory responses in the intestine are pertinent to intestinal barrier.^37–40^ In this study, fluorescent in situ hybridization (FISH) experiments showed an increase in bacteria adhering to colonic epithelial cells in the crypt base following 1.2 ~ 5 mg/kg of S-amlodipine treatment (Figure 5(a)), suggesting that colonic barrier function might be altered in rats, because invasion of opportunistic pathogens likely disrupts the integrity of the intestinal mucosa. Subsequently, we examined the colonic barrier integrity and found that 1.2 ~ 5 mg/kg of S-amlodipine treatment damaged the colonic barrier, leading to increased intestinal permeability in rats. As shown in Figure 5(b), the colonic histology images showed the loss of partial mucosal epithelial and intestinal gland cells with a high histological score in the S-amlodipine group compared to the control group, suggesting structural damage to the intestines in the S-amlodipine-treated rats. 5 mg/kg of S-amlodipine administration noticeably shortened the length of colon tissues (Figure S7), and markedly decreased the mRNA and protein levels of barrier-associated tight junctional proteins, such as Claudin-4 and Occludin in the colon of S-amlodipine-treated rats compared with the control rats (Figure 5(c,d)). Immunohistochemical staining results indicated that the expression of barrier-associated tight junctional protein Claudin-3 was decreased in the colon of the S-amlodipine group (S-AM-1.2 and S-AM-5) (Figure 5(e)), which confirmed the damaged intestinal barrier in the colon of rats. Moreover, we observed a reduction in intestinal mucus secretion in the S-amlodipine group as evidenced by AB-PAS staining (Figure S8). The intestinal mucus plays a crucial role in maintaining intestinal barrier integrity. This decreased mucus secretion further supports the observation of intestinal barrier damage following 1.2 ~ 5 mg/kg of S-amlodipine treatment. Subsequently, we observed that S-amlodipine treatment intensified the intestinal permeability of rats, as indicated by the enhanced levels of fecal albumin and serum LPS (Figure 5(f)). This largely increases the risk of pathogens invading the circulatory system. However, there were no significant changes in colonic barrier integrity in the S-AM-0.6 group compared to the control group (Figure S6(b-e)). In summary, the results of this study demonstrated that S-amlodipine-induced alterations in the gut microbiome can lead to colonic barrier dysfunction and increased intestinal permeability, which poses a potential risk to rat health.

### The altered gut microbiome triggered liver inflammation and associated dysfunction by releasing LPS in bloodstream and activating LPS-TLR4 pathway in liver

**Figure 6.**
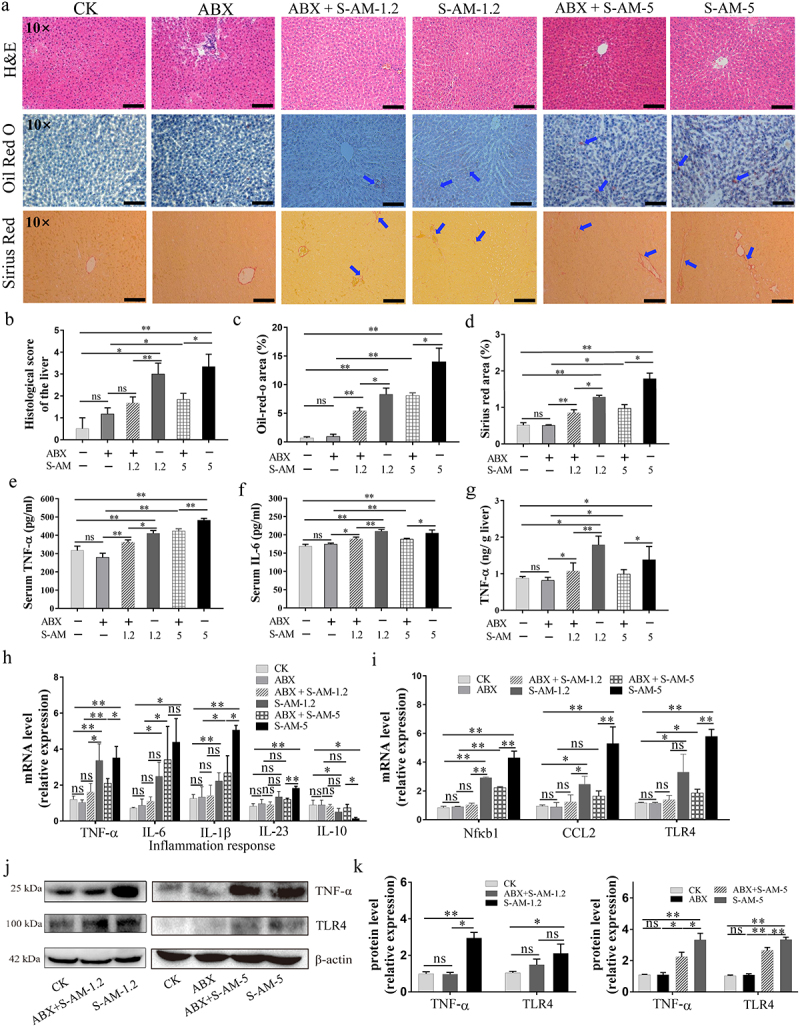
S-amlodipine-induced hepatic inflammation in rats is mediated by disordered intestinal microbiota and their LPS. (a) Representative pictures of liver sections stained with H&E, Oil Red O, or Sirius Red were obtained. Arrows indicate regions of lesions. (b) The histology score of the liver was evaluated (b), and the quantitation of the Oil-Red-O area (c) and fibrosis area was performed (d). *N* = 3 per group. Scale bars, 50 μm. (e-f) the serum levels of TNF-α (e) and IL-6 (f) were measured by ELISA. *N* = 5 per group. (g) Hepatic TNF-α level was determined. *N* = 5 per group. (h) The mRNA expression of inflammatory genes in the liver was detected by RT-qPCR. *N* = 5 per group. (i) The mRNA expression of Nfκb1, chemokine CCL2, and TLR4 genes in the liver was detected by RT-qPCR. *N* = 5 per group. (j-k) Representative images of western blot analysis on liver tissues were obtained (j), and the protein expression of TNF-α and TLR4 was quantified (k). *p < 0.05, **p < 0.01. ns, no significant difference.

**Figure 7.**
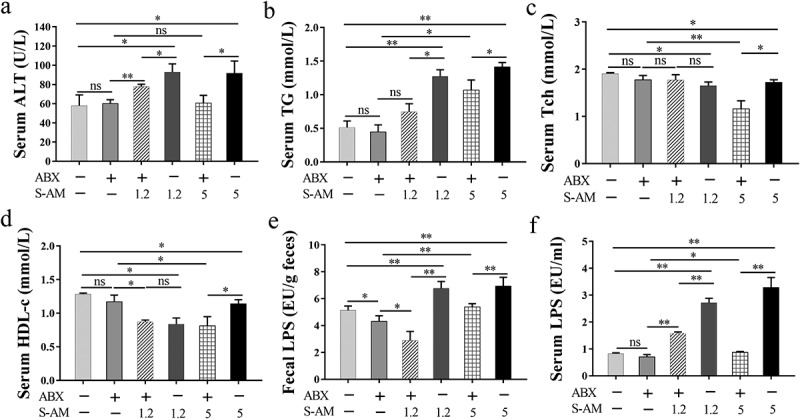
The shift of liver indexes in rats induced by S-amlodipine is mediated by disordered intestinal microbiota. (a-d) the serum levels of ALT (a), TG (b), Tch (c), and HDL-c (d) were measured in rats. *N* = 3 per group. (e) The fecal LPS level was determined. (f) The serum LPS level was measured. (e-f) *N* = 5 per group. ALT, alanine transaminase; TG, triglycerides; Tch, total cholesterol; HDL-c, high-density lipoprotein cholesterol. *p < 0.05, **p < 0.01. ns, no significant difference.

The mammalian gastrointestinal tract is considered an “exteriorized organ”, which is closely related to the liver via the gut-liver axis.^41^ We found that the relative abundance of intestinal *E. coli* and *P. excrementihomini*s increased in 1.2 ~ 5 mg/kg of S-amlodipine-treated rats, along with an increase in intestinal permeability. Once the intestinal barrier is compromised, opportunistic pathogens and their LPS may translocate to the liver via enterohepatic circulation, potentially causing liver dysfunction. To examine how the altered gut microbiota affects liver inflammation and dysfunction, rats were treated with antibiotic cocktails in drinking water to partially remove the intestinal microbiota before administering S-amlodipine, according to previous studies.^42,43^ The microbial load (copy number in feces) markedly decreased by 398-fold (log_10_ FC = 2.6) at week 9 in antibiotic-treated rats (ABX + S-AM-5 group) compared with S-amlodipine-treated rats (S-AM-5 group) (Figure S9), indicating the successful removal of a portion of the intestinal bacteria. The study observed that treatment with ABX + S-amlodipine decreased hepatocyte ballooning (H&E staining), fat deposition (Oil Red O staining) and liver fibrosis areas (Sirius Red staining) (Figure 6(a–d)). Liver inflammation in the ABX + S-amlodipine group was also alleviated, as evidenced by decreased levels of inflammatory markers (serum TNF-α and IL-6) (Figure 6(e,f)), hepatic TNF-α level (Figure 6g), and mRNA/protein expression of inflammatory cytokines and chemokines (TNF-α, IL-1β, IL-23, Nfκb1, CCL2, and TLR4) (Figure 6(h–k)). Furthermore, liver dysfunction, as indicated by markers of liver function indicators (serum ALT and TG), was attenuated in the ABX + S-amlodipine-treated rats compared with that in the S-amlodipine-treated rats (Figure 7(a,b)). However, cholesterol metabolism disorder was not alleviated in the ABX + S-AM-5 group compared to that in the S-AM-5 group, as evidenced by the decreased levels of serum Tch and HDL-c (Figure 7c,d). Importantly, the liver immunohistochemical staining results (Figure 6a–d) and the liver inflammation and function indices (Figures 6(g–k) and 7(a–d)) did not show noticeable effects in the ABX-treated group compared with the control group, suggesting that the antibiotics alone were not responsible for liver dysfunction and inflammation. Previous studies have also indicated that the rational use of broad-spectrum antibiotics does not directly affect liver disease.^44^ Additionally, studies have shown that antibiotics can have toxic effects on the liver.^45,46^ Based on these findings, it can be concluded that S-amlodipine-induced liver inflammation and dysfunction are mediated by the gut microbiota.

Metagenome sequencing analysis revealed that 1.2 ~ 5 mg/kg of S-amlodipine treatment reduced the relative abundance of beneficial bacteria, specifically *A. muciniphila* and *B. uniformis* (Figures 3(i) and S5(i)), which are known to play a role in regulating intestinal barrier integrity. Conversely, S-amlodipine treatment led to an increase in the relative abundance of opportunistic pathogens, primarily *E. coli* (Figures 3(j) and S5(j)), which is associated with LPS production. The enhanced abundance of *E. coli*, a member of the gamma-*Proteobacteria*, was likely responsible for the increased levels of intestinal LPS, as evidenced by the elevation in fecal LPS content (Figure 7(e)). Previous studies have indicated that LPS is mostly derived from *E. coli* .^47,48^ Jang et al. reported that stressor-induced enrichment of *Proteobacteria* populations, particularly *E. coli*, and subsequent LPS production caused gastrointestinal inflammation in mice.^49^ Therefore, *E. coli* is considered a contributor to the increase in intestinal LPS levels. Additionally, *P. excrementihominis*, another opportunistic pathogen, has been found to be strongly associated with intestinal metabolites,^50^ which might be related to the increased levels of intestinal LPS. In this study, the reduced abundances of *A. muciniphila* and *B. uniformis* were potentially associated with increased serum LPS levels. It has been reported that *A. muciniphila* and *B. uniformis* can decrease the level of serum gut-derived LPS by regulating intestinal barrier function.^51,52^ This implied that 1.2 ~ 5 mg/kg of S-amlodipine administration increased both intestinal and serum LPS levels, which might be mostly ascribed to the enriched *E. coli* and decreased *A. muciniphila* and *B. uniformis* in the gut of rats.

**Figure 8.**
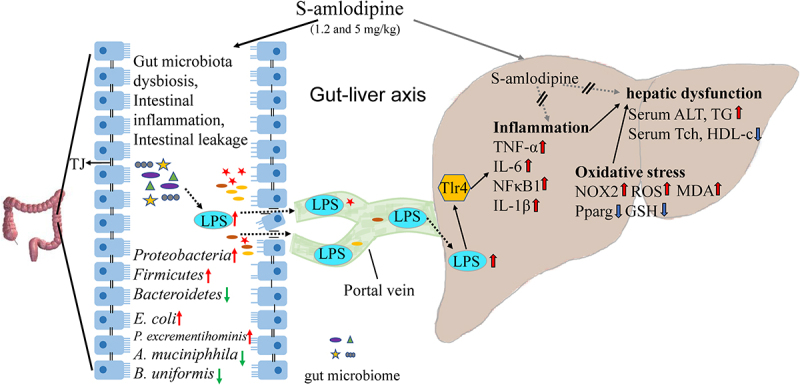
Schematic summary. S-amlodipine does not directly cause hepatocytes damage. However, 1.2 ~ 5 mg/kg of S-amlodipine treatment disrupts the gut microbiome in rats by reducing the abundance of beneficial bacteria and increasing the abundance of opportunistic pathogens. This alteration leads to an increase in fecal LPS content, triggering intestinal inflammation and upregulating the mRNA and proteins expression of pro-inflammation cytokines. The increased intestinal permeability results in the downregulation of tight junction proteins, and subsequently increased serum LPS concentration. Bacteria-derived LPS enters the liver through the portal vein and binds to TLR4 on hepatocytes and immune cells, inducing liver inflammation and oxidative stress. This process ultimately gives rise to hepatic inflammation and associated dysfunction in rats.

Mounting evidence has shown that enrichment of gut bacterial LPS is associated with the onset and progression of liver inflammation, which plays a vital role in the pathogenesis of liver disease.^53–56^ LPS can bind with the LPS-binding protein (LBP)-CD14 complex to activate TLR4, triggering a crucial inflammatory cascade in NAFLD, which is generally accepted in previous investigations.^57–59^ In this study, treatment with ABX + S-amlodipine considerably diminished the accumulation of fecal and serum LPS (Figure 7(e,f)), leading to reduced inflammatory responses and ameliorated liver inflammation in ABX + S-amlodipine-treated rats compared with S-amlodipine-treated rats. Our novel findings that S-amlodipine-induced elevation of fecal LPS content, along with a compromised intestinal barrier, facilitated the entry of gut-derived LPS into the liver through the blood circulation system to bind with TLR4 in liver immune cells, upregulated the expression of proinflammatory cytokines and chemokines, and finally induced liver inflammation in rats (Figure 8).

To further confirm the role of LPS in liver inflammation, *in vitro* hepatocyte cultures were conducted. As expected, we found that treatment with 3.5 EU/ml of LPS (serum LPS reference concentration in normal rats after 5 mg/kg of S-amlodipine treatment, Figure 7(f)) significantly caused hepatocytes inflammation and part of hepatocytes injury (Figure S10). This was evidenced by increased levels of TNF-α (an indicator of liver inflammation), ALT (an indicator of liver function), and Oil-red-o areas in BRL hepatocytes compared to treatment with 0.9 EU/ml LPS (serum LPS reference concentration in control rats) (Figure S10(a,c,e)). Notably, treatment with 1 EU/ml LPS (serum LPS reference concentration in ABX-treated rats after 5 mg/kg of S-amlodipine treatment, Figure 7(f)) remarkably alleviated hepatocyte inflammation by decreasing the levels of TNF-α and ALT in BRL hepatocytes compared to treatment with 3.5 EU/ml LPS (Figure S10(a,c)). Additionally, 1 EU/ml of LPS treatment markedly mitigated part of the hepatocyte injury by decreasing the Oil-Red-O areas compared to 3.5 EU/ml of LPS (Figure S10(d,e)). However, the malonaldehyde (MDA) level in BRL hepatocytes was no significant difference under the range of 0.9 ~ 3.5 EU/ml LPS treatment (Figure S10(b)). These *in vitro* experimental results were consistent with the findings *in vivo*, supporting the conclusion that S-amlodipine-induced enrichment of gut-derived LPS causes liver inflammation. Collectively, these data demonstrated that S-amlodipine-induced alterations in the composition of the gut microbiome and increased production of gut-derived LPS ultimately caused hepatic inflammation and dysfunction in rats

## Discussion

In recent decades, hypertension has been recognized as a key cause of cardiovascular disease and untimely decease worldwide.^60^ The antihypertensive medication S-amlodipine has gained widespread use in clinical practice owing to its high efficacy. Although few studies have reported the side effects of S-amlodipine in hypertensive patients, clinicians have advised caution when prescribing S-amlodipine to hypertensive patients with existing liver dysfunction. However, it is not known whether long-term treatment with S-amlodipine can cause adverse effects in the liver. This study is the first to demonstrate that administration of 1.2 ~ 5 mg/kg of S-amlodipine for a period of 7 weeks caused the enrichment of *E. coli* and the decline of *A. muciniphila* and *B. uniformis*, the enhancement of enteric bacteria-derived LPS and intestinal permeability, and subsequently LPS accumulation in the liver, leading to hepatic inflammation and associated dysfunction in rats (Figure 8).

Emerging evidence has shown that the gut microbiota and bacterial endotoxin (LPS) are essential mediators of human health and disease, particularly in intestinal and hepatic conditions through the gut-liver axis.^47,52,61,62^ In the present study, we revealed that S-amlodipine-induced liver dysfunction was mediated by the intestinal microbiota and LPS by combining an *in vivo* rat experiment with an *in vitro* hepatocytes culture experiment. S-amlodipine did not cause damage to hepatocytes *in vitro*, indicating that the liver dysfunction observed after S-amlodipine treatment was not due to the direct toxicity of S-amlodipine. The levels of LPS in both feces and serum were significantly reduced, and hepatic inflammation was alleviated in gut microbiota-deleted rats, suggesting that the gut microbiota and their LPS play important roles in S-amlodipine-induced liver inflammation. Furthermore, *in vitro* LPS-induced BRL hepatocyte experiments verified that LPS-mediated liver damage and treatment with a serum LPS reference concentration (after 5 mg/kg of S-amlodipine treatment) significantly caused hepatocellular inflammation by increasing the levels of TNF-α and the liver function indicator ALT in BRL hepatocytes.

Our results also revealed that S-amlodipine treatment triggered an intestinal inflammatory response and increased the intestinal permeability in rats. In the case of increased intestinal permeability, gut-derived LPS could enter the portal circulation and subsequently be recognized by the TLRs complex in the liver, which could cause liver inflammation.^23,63,64^ In this study, we verified that gut-derived LPS enters the liver through enterohepatic circulation and triggers a battery of inflammatory responses, resulting in liver inflammation and dysfunction. Although we found that 1.2 ~ 5 mg/kg of S-amlodipine induced enrichment of LPS-mediated liver inflammation, liver inflammation was of low degree and predominantly localized in the periportal compartment compared to conditions such as alcoholic liver disease or nonalcoholic fatty liver disease (NAFLD), because hepatocyte ballooning and fat deposition in the liver tissue were unevenly distributed.

It is well known that LPS plays an important role in directly or indirectly regulating gut integrity and systemic host homeostasis. LPS, which is mainly derived from the gut microbiota, consists of a lipid structure and polysaccharide components located in the outer membrane of gram-negative bacteria. In this study, we found that 1.2 ~ 5 mg/kg of S-amlodipine caused significant changes in the relative abundances of *E. coli*, *A. muciniphila* and *B. uniformis*, which could be linked to increased levels of LPS in both the intestine and serum. *E. coli* has been reported to release LPS and induce liver inflammation in germ-free mice colonized with *E. coli* fed a high-fat diet,^65^ which was achieved through modulation of the LPS-TLR4 signaling pathway. We observed that *E. coli* was significantly enriched in the S-amlodipine-treated group, which may be accompanied by an increase in LPS in the intestine and serum. Interestingly, we found that *P. excrementihominis* was remarkably enriched in the S-amlodipine-treated group, but whether it is associated with increased intestinal LPS remains unknown. In addition, several studies have suggested that probiotics, such as *A. muciniphila* and *B. uniformis* have anti-inflammatory properties that contribute to maintaining the integrity of the gut barrier, and can reduce the translocation of bacteria-derived LPS from the gut into the bloodstream in mice.^9,27,51,52^ In our study, the relative abundances of *A. muciniphila* and *B. uniformis* were markedly decreased in the S-amlodipine-treated group, which was likely related to increased serum gut-derived LPS levels. A Previous study (PMID: 34862599) reported that amlodipine aspartate significantly alleviated NAFLD and hypertension by modulating gut microbiota, including enhanced *Akkermansia*, in mice with NAFLD and hypertension.^66^ Our results diverge from that in the mentioned report due to variations in animal models and drugs utilized. Unlike the previous study,^66^ which used mice model with NAFLD and hypertension, our study utilized healthy Sprague Dawley (SD) rats. Moreover, we employed S-amlodipine treatment for rats, whereas the previous study utilized amlodipine. Consequently, distinct drugs manifest diverse effects on different gut microbiota (mice with NAFLD and hypertension and healthy rats). Collectively, our study revealed that the altered gut microbiome and LPS play a significant role in liver inflammation and dysfunction induced by S-amlodipine in rats. However, whether these effects of S-amlodipine really occur in humans require further confirmation.

Although amlodipine is generally considered to be well tolerated in clinical practice, the four reported cases of amlodipine-induced liver damage and inflammation^6,7,67,68^ warrant retrospective evaluation to determine whether amlodipine-induced liver damage is associated with dysbiosis of gut microbiota. We initially employed healthy SD rats for experimental design because the composition of intestinal microbiota indicated by the *Firmicutes*/*Bacteroidetes* (F/B) ratio (a potential biomarker for gut dysbiosis) is more similar to that of hypertensive patients. The F/B ratio is reported to be 0.46 in hypertensive patients (*n* = 99) and 0.66 in healthy individuals (*n* = 41).^69^ The F/B ratio is approximately 0.49 in healthy SD rats, and 22.5 in spontaneously hypertensive rats (SHRs).^70^ Given the similarity in the F/B index between hypertensive patients and SD rats, we initially employed SD rats for experimental design to illustrate S-amlodipine-induced the changes of gut microbiota caused liver inflammation. However, further studies are warranted to include the rats of SHR model to compare whether the gut microbiota and associated liver inflammation are differences between the SD and SHR rat models.

In this study, we demonstrate that S-amlodipine induced significant alterations of gut microbiota, leading to gut dysbiosis, which subsequently activated LPS-derived liver inflammation. Previous studies reported a different mechanism by which amlodipine exhibits beneficial modulation in the context of paracetamol-induced liver inflammation in Wistar rats (PMID: 29441826; PMID: 27829805).^71,72^ However, the lack of gut microbiota analysis in previous studies hinders comparability with our study. Notably, it has been observed that paracetamol can induce gut dysbiosis in murine models.^73–75^ Consequently, the initial state of Wistar rats with paracetamol-induced liver inflammation in both previous studies potentially experienced gut dysbiosis, and subsequent treatment with amlodipine potentially complicated the status of their gut microbiota. Regrettably, previous studies did not investigate changes in gut microbiota under amlodipine medication.^71,72^ Therefore, it is challenging to determine whether liver inflammation aggravated or alleviated following an intricate change of intestinal microbiota under influence of amlodipine.

In summary, we found that 1.2 ~ 5 mg/kg of S-amlodipine treatment caused alterations in gut microbiota, resulting in both intestinal inflammation and subsequent liver dysfunction in a rat model. However, little attention has been paid to the clinical safety of S-amlodipine in individuals. Nevertheless, clinicians recommend caution when administering S-amlodipine to hypertensive patients with liver dysfunction. Further studies are needed to determine whether S-amlodipine can induce liver dysfunction in the general population. It is worth noting that this study employed healthy rats. Further investigation is warranted to determine if similar results can be observed in clinical experiments involving SHRs.

## Conclusions

Collectively, the results of our study demonstrated that oral administration of 1.2 ~ 5 mg/kg of S-amlodipine led to liver inflammation and dysfunction in rats, which was induced by activation of the LPS-TLR4 pathway in the liver. The increased concentration of LPS in the bloodstream is associated with S-amlodipine-induced alterations in the gut microbiome, characterized by an increase in *E. coli* and a decrease in probiotics such as *A. muciniphila* and *B. uniformis*. The altered gut microbiome disrupts the intestinal barrier, resulting in the leakage of LPS into the bloodstream. Our study highlights the potential adverse effects of S-amlodipine on the liver by altering the gut microbiome. A cohort study in a clinical setting is needed for further exploration.

## Materials and methods

### Animal experimental design

Seven-week-old male rats (SPF, *N* = 40) were purchased from Beijing Huafukang Biotechnology Co., Ltd. and housed in a specific-pathogen-free environmentally controlled room with a 12-h light–dark cycle and all the rat had free access to water and food. After acclimation for 1 week, all rats were randomly divided into eight groups (*N* = 5 per group), including CK group (normal feed (ND)), ABX group (ND + antibiotic cocktails (0.2 g/L metronidazole, 0.1 g/L vancomycin, 0.2 g/L ampicillin, and 0.2 g/L neomycin)), ABX + S-AM-0.6 group (antibiotic cocktails + 0.6 mg/kg of S-amlodipine + ND), ABX + S-AM-1.2 group, ABX + S-AM-5 group, S-AM-0.6 group (0.6 mg/kg of S-amlodipine + ND), S-AM-1.2 group (1.2 mg/kg of S-amlodipine + ND), and S-AM-5 group (5 mg/kg of S-amlodipine + ND), as shown in Figure 1. S-amlodipine was dissolved in 0.2% sodium carboxymethylcellulose aqueous solutions, and antibiotic cocktails were dissolved in sterile water. The rats in the S-AM and ABX + S-AM groups were administrated corresponding dose of S-amlodipine (0.6 ~ 5 mg/kg) once daily for 7 weeks by oral gavage, and the same dose of placebo was administered to the CK and ABX groups using the same procedure. To understand the role of gut flora in S-amlodipine-induced liver inflammation and dysfunction, rats (*N* = 20, ABX and ABX + S-AM groups) were administrated antibiotic cocktails for a week, and the rats were given antibiotic cocktails for one week every two weeks until the end of the experiment, to minimize the number of gut bacteria. Rats were euthanized by cervical dislocation, feces, blood, colon and liver tissues were collected for follow-up mechanism analysis, and all rats and liver tissues were weighed. All animal procedures were approved by the Committee on the Ethics of Animal Experiments of Nankai University (Project IRM-DWLL-2020125).

### Reagent and fecal collections

S-amlodipine was purchased from Beijing DehangWuzhou Technology Co., Ltd. Metronidazole, vancomycin, ampicillin, and neomycin were obtained from Beijing Solarbio Biotechnology Co. Ltd. HepG2 and BRL cells were obtained from Procell Life Science & Technology Co. Ltd. LPS derived from *E. coli* O55:B5 (L2880; Sigma) was purchased from Sigma-Aldrich Co., Ltd.

At weekly intervals, the rats were kept in an empty cage without bedding for 20 min to collect fresh stool samples into sterile 4 ml centrifuge tubes, which were rapidly transferred to lab and stored at −80°C until analysis.

### DNA extraction and metagenomic sequencing

Genomic DNA was extracted from 200 mg of stool samples using the Stool Genomic DNA Extraction Kit (D2700, Solarbio) according to the manufacturer’s protocol. Briefly, 800 μL SA buffer was added to the fecal sample and placed on ice for 10 min. The pellets were homogenized by adding 400 μL SB buffer, RNase A and proteinase K, and then the mixture was heated in 65°C water bath for 1 h. 600 μL of SC buffer was added to the supernatant, and the bacterial DNA was eluted by using an adsorption column. DNA concentration was determined using a Nanophotometer® N60 (Implen, Germany), and the quality of the bacterial DNA was detected by agarose gel electrophoresis. DNA libraries were constructed using 1 μg of DNA and subsequently sheared using an ultrasonic cleaner (BILON3-120B, Shanghai) for 290 s. Sheared genomic DNA was purified with 0.8 × Superior AMPure XP Alternative (12601ES08, Yeasen) and end repair, dA-tail, adaptor ligation, AMPure XP bead purification, PCR enrichment, and PCR product purification were performed using the DNA Library Prep Kit (Novogene) with minor modifications to the manufacturer’s protocol. Whole-metagenome shotgun sequencing was performed on twenty-three fecal samples using the Illumina HiSeq 2500 platform. The obtained paired reads were trimmed, and the rat genome was subsequently removed from the trimmed reads using bowtie2. The clean reads obtained by bowtie2 were compared with the sequence information in known databases to determine the relative abundance of species, which was analyzed using MetaPhlAn2.^76^

### Histopathological examination, immunohistochemical staining and fluorescent in situ hybridization (FISH)

The Fresh colon and liver tissues were promptly fixed in 4% paraformaldehyde solution (G1101, Servicebio) and embedded in paraffin. Colon and liver tissues were sectioned and stained with H&E. Liver slides were stained with Oil Red O and Sirius Red. The stained section samples were placed under an advanced positive fluorescence microscope (Axio Imager Z1, ZEISS) for observation, and a representative image of five fields per sample is shown. The degree of colon damage was evaluated in accordance with a report by Moreels et al.’s report.^77^ Similarly, we evaluated the degree of liver damage according to the NAFLD activity score system.^78^ The Oil Red O- and Sirius Red-positive areas were quantified using ImageJ software.

The colonic sections were stained with primary antibodies (1:100) against Claudin-3 (Servicebio, GB14069, Wuhan), which was used to analyze the expression of Claudin-3.

Colon tissues were immediately fixed using in situ hybridization fixative and embedded in paraffin. The sections were deparaffinized, digested, and washed with PBS. Pre-hybridization solution was added to each section and incubated for 1 h at 37°C. After pre-hybridization solution was removed, the Cy3-conjugated bacterial probe (EUB338, General Biol, China) hybridization solution was added the sections and incubated overnight at 37°C in a dark humidity chamber. Next, the sections were washed and counterstained with DAPI for 8 min in the dark and mounted using mounting medium.

### RNA isolation, real-time qPCR analysis and whole-genome RNA sequencing

RNA was extracted from the colon and liver using TRNzol Universal Reagent (TIANGEN) according to the manufacturer’s instructions. RNA quality was determined using a Nanophotometer® N60, and RNA integrity was evaluated by 2% agarose gel electrophoresis. Library preparation for RNA sequencing was conducted using the TruSeq^TM^ RNA sample preparation kit (Illumina), and the library was sequenced using an Illumina NovaSeq 6000 sequencer at Shanghai Majorbio Medicine Technology Co., Ltd. To obtain clean data, the raw data were quality controlled using SeqPrep (https://github.com/jstjohn/SeqPrep), and the adapters were removed using Majorbio (Shanghai). The clean reads were aligned to a reference genome (GCF_015227675.2) using the HISAT2.^79^ Differentially expressed genes (DEGs) were determined using DESeq,^80^ and DEGs with a P-adj of ≤ 0.05 and |log2 fold change| of ≥ 1 were considered to be significantly differentially expressed. The Kyoto Encyclopedia of Genes and Genomes (KEGG) pathways were further analyzed using path view.^81^

Real-time qPCR was performed with TB green reagent (Takara) using qPCR equipment (LightCycler96, Roche), and NADPH as a housekeeping gene. All primer sequences used for RT-qPCR are listed in the supplementary materials (Table S1). The process of qPCR was performed as follows: initial denaturation for 30 s at 95°C, followed by 40 cycles of 5 s at 95°C and 30 s at 60°C, melting for 10 s at 95°C, 60 s at 65°C, and 1 s at 97°C, and then 37°C for 30 s. Relative gene expression was calculated using the ΔΔCT method with NADPH as a housekeeping gene control.^65^

### Western blot analysis, biochemical test and enzyme–linked immunosorbent assay (ELISA)

The liver or colon (100 mg) was chopped and suspended in RIPA buffer (#9806, CST) with 1× protease inhibitor cocktail (P8340, Sigma-Aldrich), and then the mixture was homogenized in a tissue grinder (JXFSTPRP-12/16, TUOHE, Shanghai). Total tissue lysates were separated by sodium dodecyl sulfate-polyacrylamide gel electrophoresis and transferred onto a nitrocellulose membrane. The membranes were incubated with primary antibodies against Claudin-4 (Proteintech 16,195–1-AP, Chicago), Occludin (Servicebio, GB11149, Wuhan), TNF-α (Proteintech 17,590–1-AP, Chicago), NF-κB (Servicebio, GB11997, Wuhan), and TLR4 (Servicebio, GB11519, Wuhan). β-Actin was used as a loading control. HRP-conjugated secondary antibody was then added to the membranes. The Immobilon® Western Chemiluminescence Detection Kit (Millipore) was used to visualize the blots.

Liver function indices (serum alanine transaminase (ALT), triglycerides, high–density lipoprotein cholesterol (HDL-c), and total cholesterol (Tch)) were analyzed using a blood biochemical analyzer (Mindray BS–2000 M, Shenzhen, China) according to the manufacturer’s instructions. The levels of proinflammatory cytokines (TNF-α and Interleukin-6 (IL-6)), malonaldehyde (MDA), and oxidative stress factors (GSH and ROS) in the liver were determined using the corresponding ELISA kit from Jiangsu Mei Biao Biological Technology Co., Ltd. Similarly, the levels of serum TNF-α, IL–6 and LPS, and fecal albumin levels were determined using the corresponding ELISA kit in accordance with the manufacturer’s protocols. All experiments were conducted in triplicate.

### Flow cytometry

The Immune cells of rat colon were isolated after digestion with collagenase IV and neutral protease for 40 min at 37°C. The mixtures were homogenized and passed through a filter screen, and then washed successively. The Percoll solution was used to separate the cells in a gradient, and the target cells were obtained by centrifugation. For surface staining, the immune cells were incubated with anti-rat CD4-FITC at 4°C for 30 min. For intracellular staining, cells were fixed and permeabilized with Fixation Buffer and Permeabilization Wash Buffer (eBioscience, Invitrogen), respectively. The Cells were stained with FoxP3-PE and IL-17a-APC at 4°C for 90 min, respectively. FACSCalibur (BD Biosciences) and FlowJo software were used to screen and analyze target-stained cells, respectively. All fluorescence-conjugated antibodies were purchased from BioLegend.

### Cell culture, Oil-Red-O staining and ELISA

HepG2 and BRL cells were cultured in Dulbecco’s modified Eagle’s medium (DMEM, Gibco, CHN) with 10% fetal bovine serum (FBS, BI, USA) and 1% double antibody (penicillin/streptomycin, Gibco, CHN) at 37°C under 5% CO_2_, respectively. Cells cultured for 2–3 generations were chosen for *in vitro* experiments. In brief, HepG2 cells were cultured for 24 h in 12-well plates, and treated with 0 ~ 20 μm S-amlodipine (dissolved on DMEM with 1% FBS) for 3 weeks. BRL cells were cultured for 24 h in 12-well plates, and treated with 0–3.5 EU/ml LPS (dissolved on DMEM with 1% FBS) for 24 h. The S-amlodipine-treated, LPS-treated, and non-treated cells were fixed with ORO Fixative for 25 min, and the Fixative was discarded. The cells were washed for two times with distilled water and soaked for 20–30 s with 60% isopropanol. The cells were stained with Oil-Red-O dye solution (1 mg/ml) for 20 min at 37°C in incubator. Then, the staining solution was discarded and rinsed for 20–30 s with 60% isopropanol, and the cells were washed for four times with distilled water. The cells were stained with Mayer hematoxylin solution for 1–2 min, and washed four times with distilled water. Stained cells were imaged using an Olympus microscope (IX53, Olympus, Japan). The Oil-Red-O positive area was quantified using ImageJ software. Cell viability was determined using CCK-8 kits, and TNF-α in the cell supernatant and intracellular ALT, MDA, and GSH levels were determined using ELISA kits.

### Statistical analysis

GraphPad Prism (version 7.0), RStudio (4.0.3) and Origin 2018 were applied for calculating the mean ± SD. SPSS Statistics (version 25.0) was used for data analysis. One-way ANOVA and independent-sample t tests were used to assess significant differences, and differences with *p* < 0.05 were considered to statistically significant.

## Supplementary Material

Supplemental Material

Supplementary file 1 clean.docx

Supplementary file 3.xls

Supplementary file 2.xls

## Data Availability

The clean sequencing datasets of fecal metagenome sequencing have been deposited in the CNCB Genome Sequence Archive (GSA) under the BioProject accession number PRJCA005398. The RNA sequence datasets of the liver were accessible under the BioProject accession number PRJCA005400.
