# Supplementary figures and images for: S-amlodipine induces liver inflammation and dysfunction through the alteration of intestinal microbiome in a rat model
Source: Gut Microbes. 2024 Feb 24;16(1):2316923. doi: 10.1080/19490976.2024.2316923 (PMC10896145; doi:10.1080/19490976.2024.2316923)

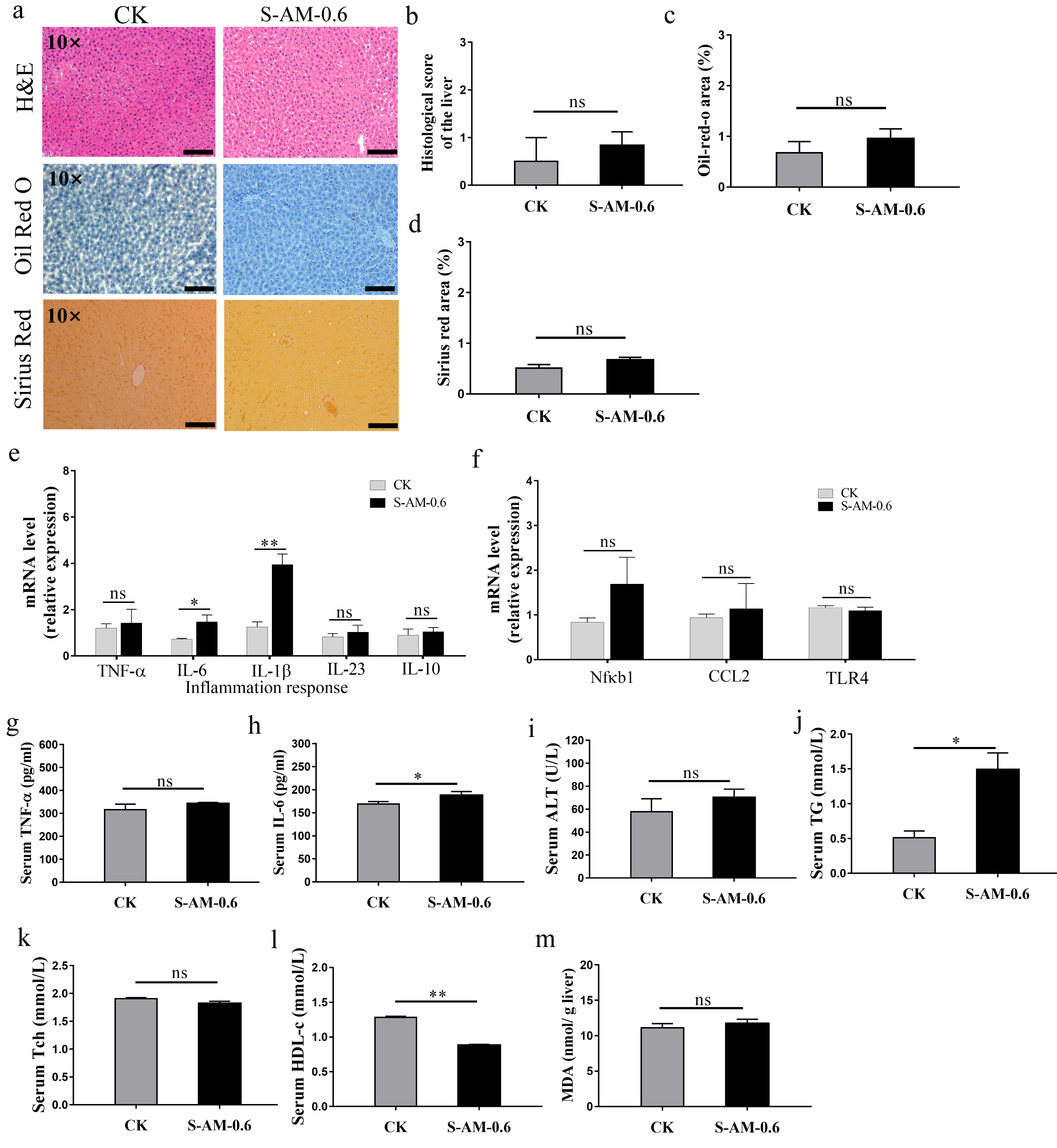

Supplement: Supplemental Material [file KGMI_A_2316923_SM4468.zip › Figure S1.tif]

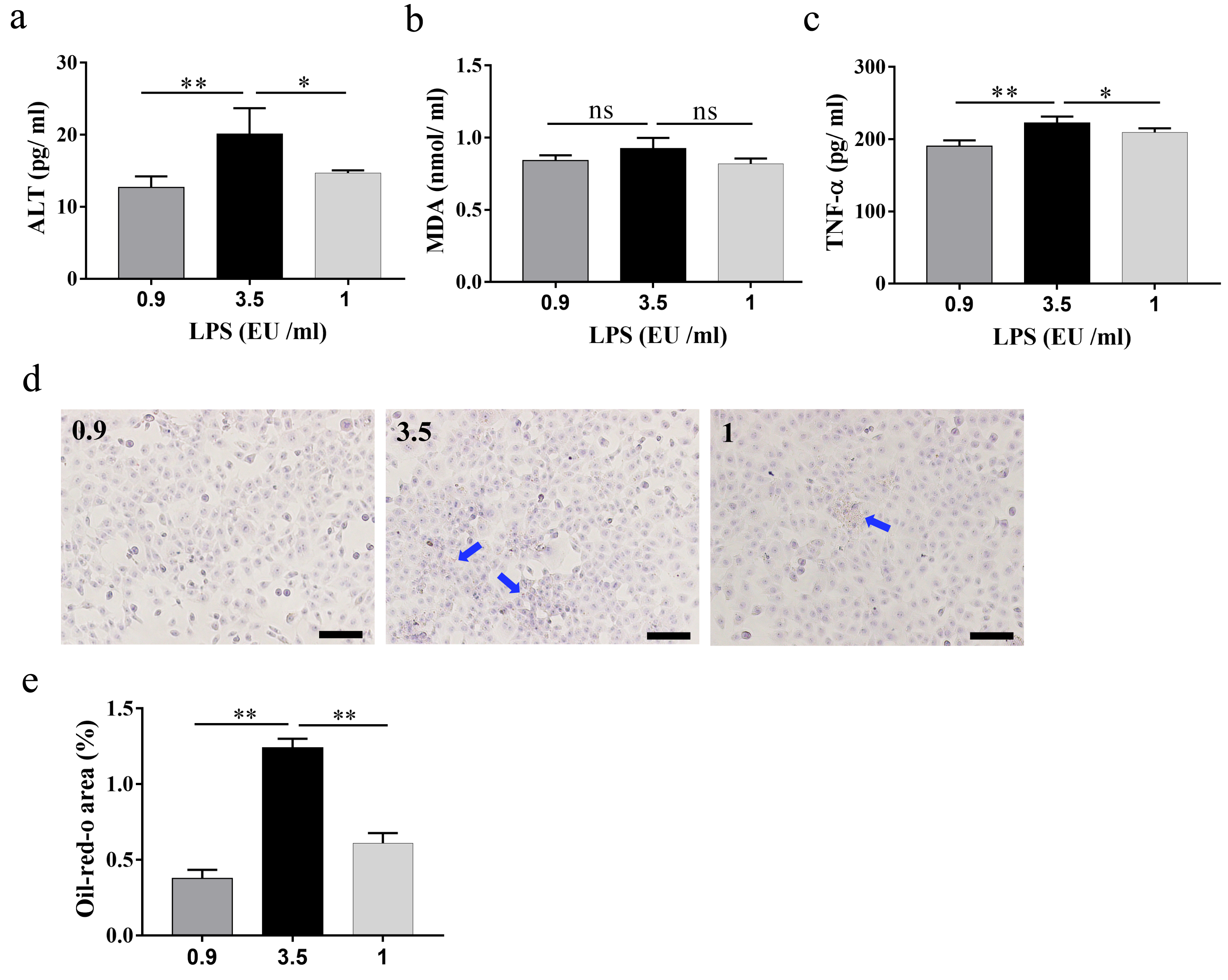

Supplement: Supplemental Material [file KGMI_A_2316923_SM4468.zip › Figure S10.tif]

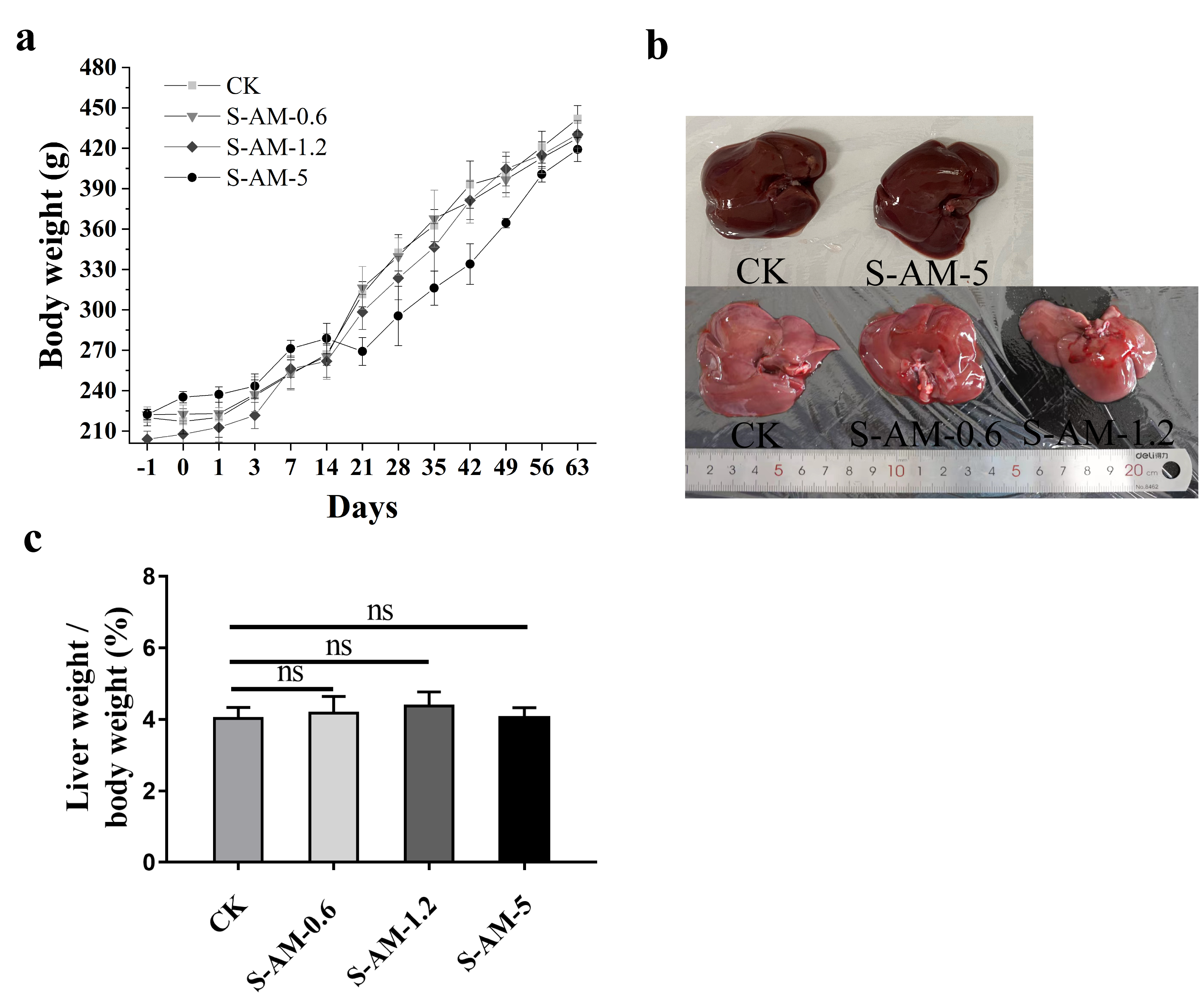

Supplement: Supplemental Material [file KGMI_A_2316923_SM4468.zip › Figure S2.tif]

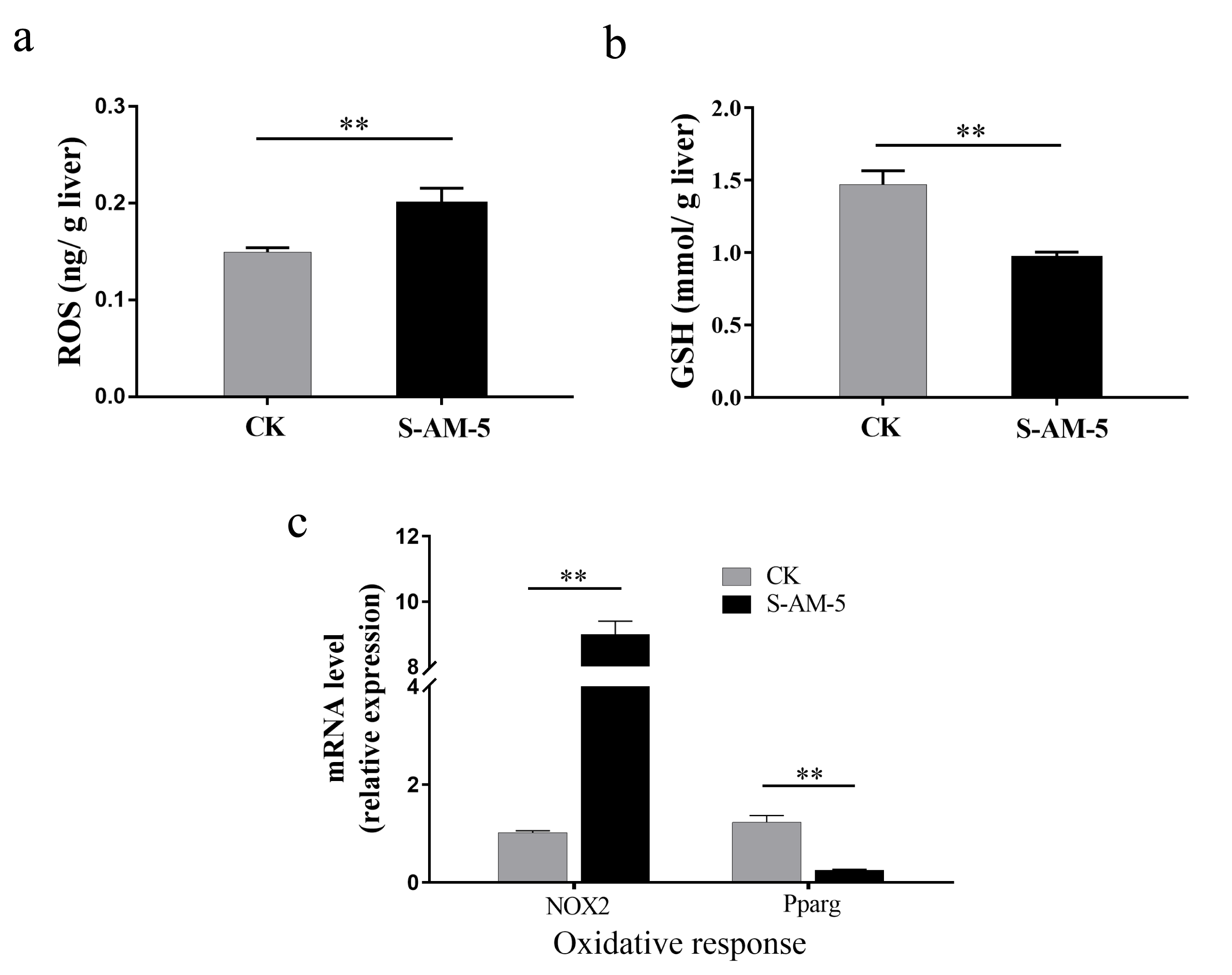

Supplement: Supplemental Material [file KGMI_A_2316923_SM4468.zip › Figure S3.tif]

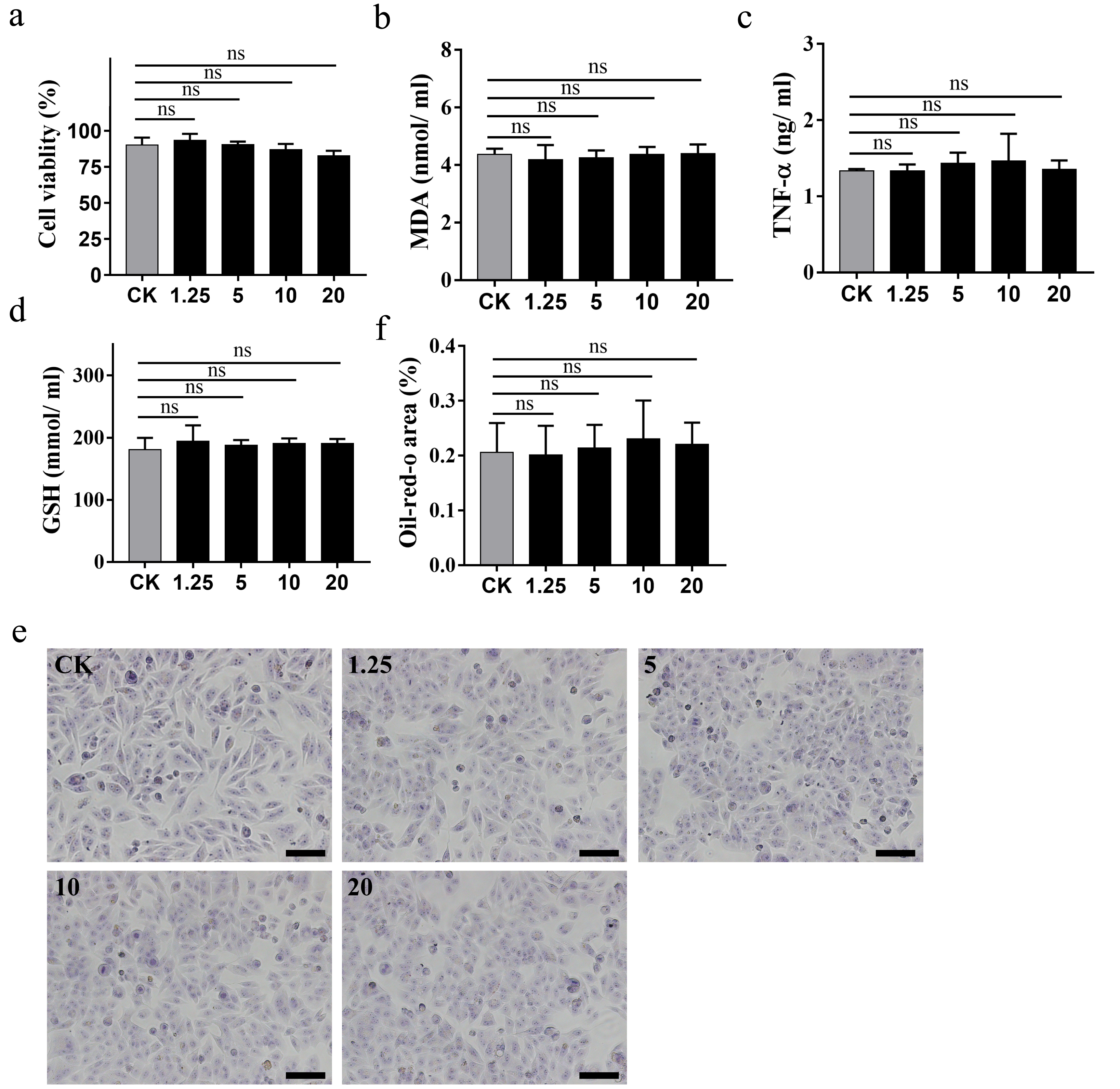

Supplement: Supplemental Material [file KGMI_A_2316923_SM4468.zip › Figure S4.tif]

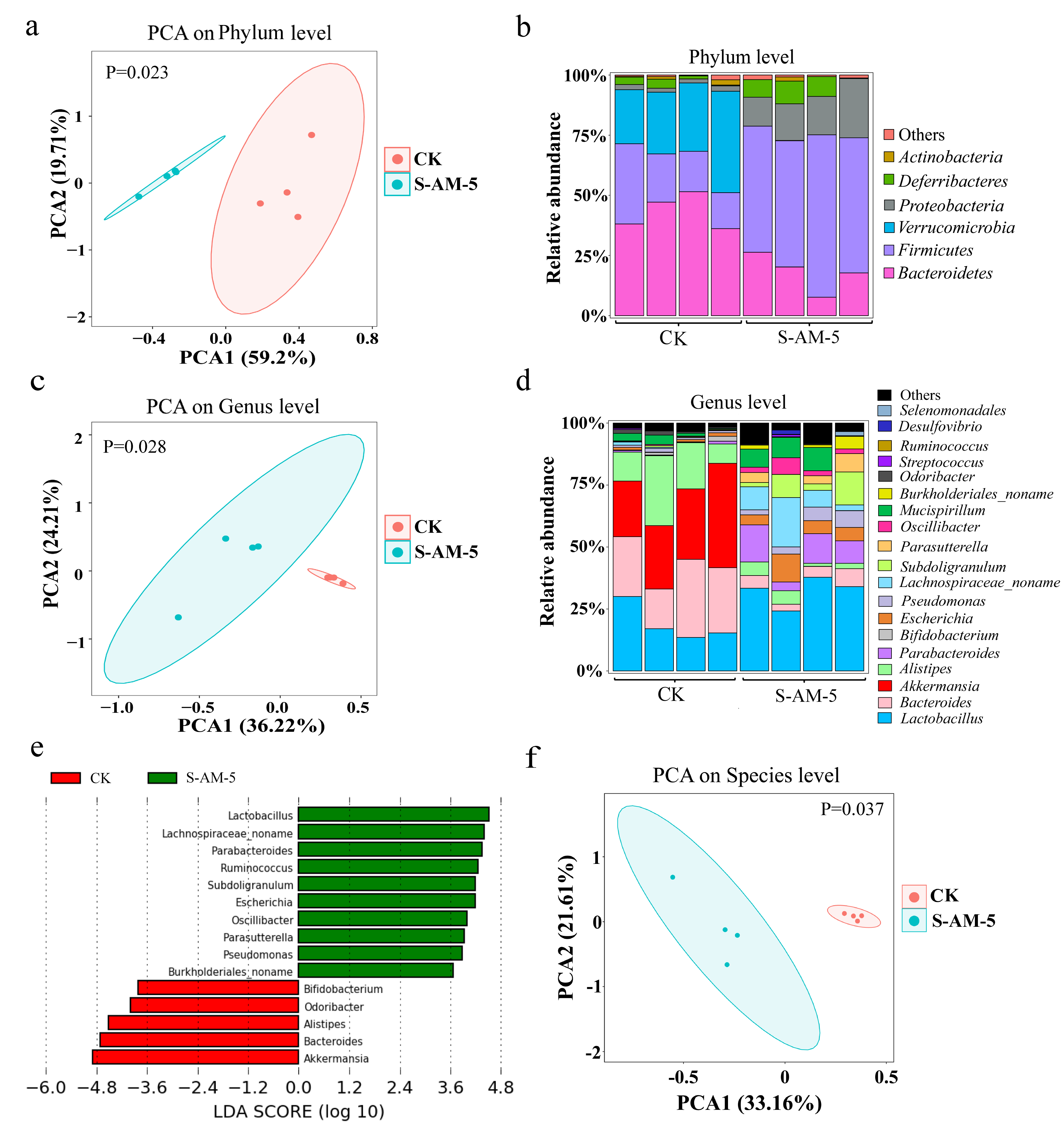

Supplement: Supplemental Material [file KGMI_A_2316923_SM4468.zip › Figure S5_1.tif]

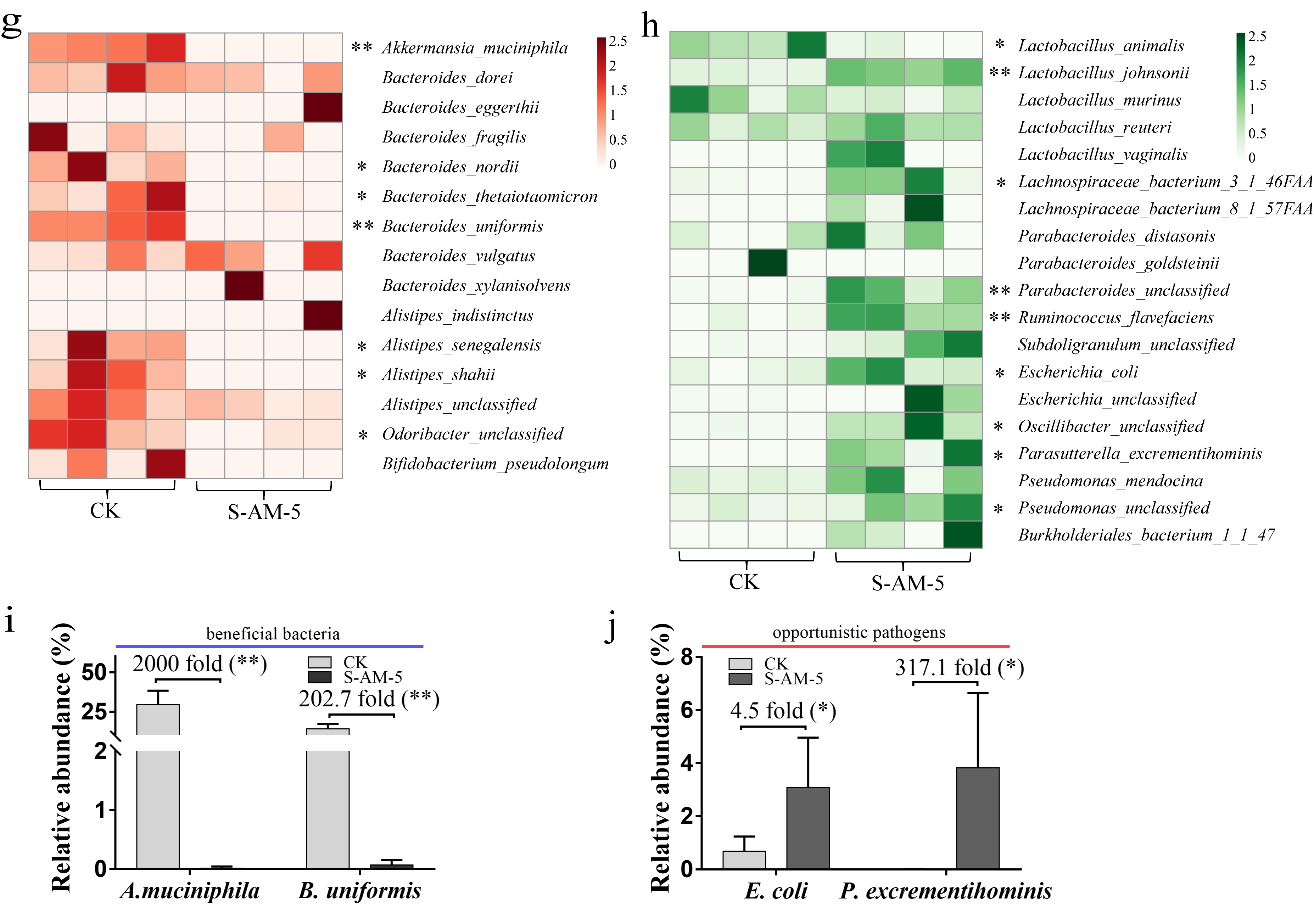

Supplement: Supplemental Material [file KGMI_A_2316923_SM4468.zip › Figure S5_2.tif]

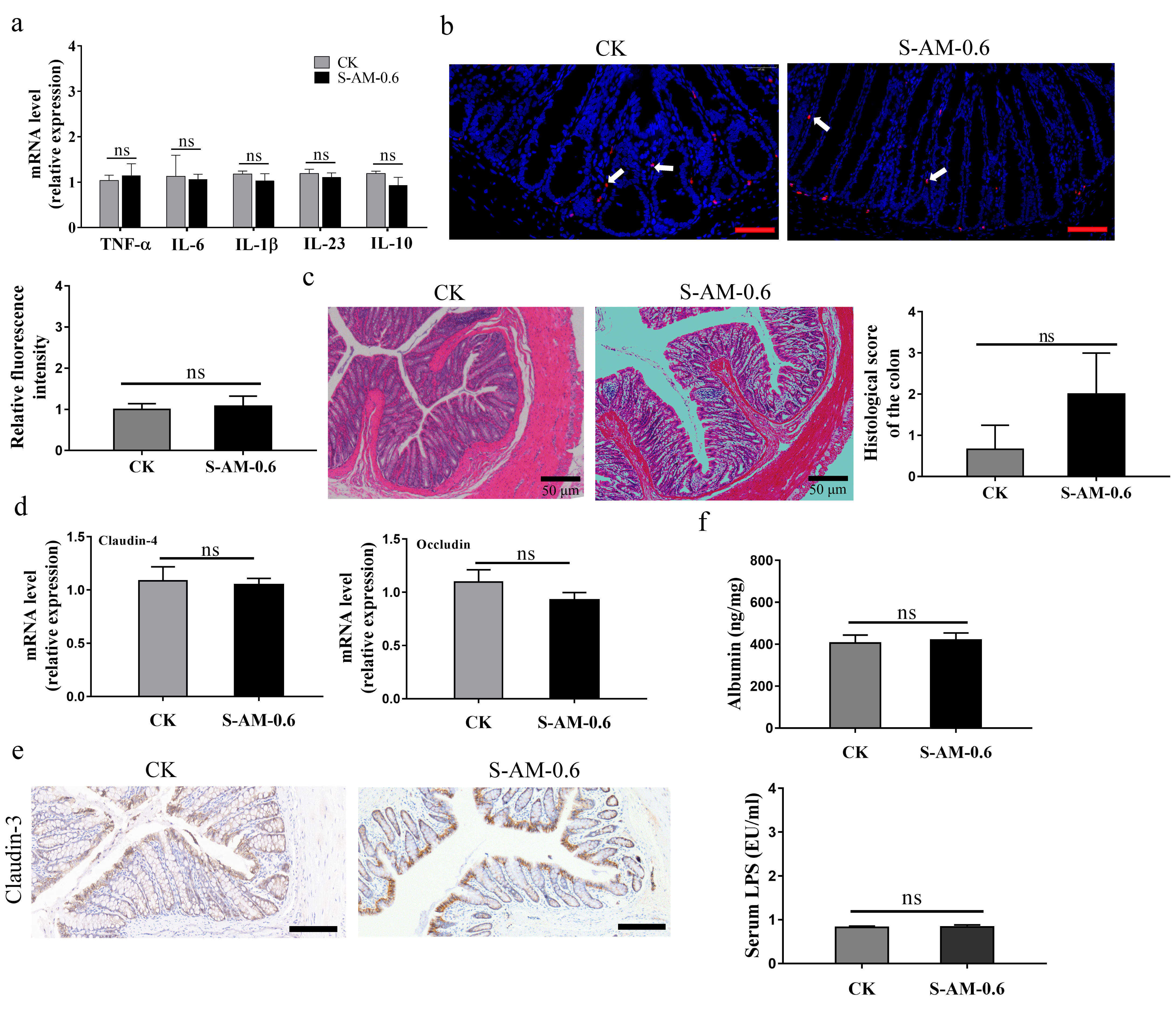

Supplement: Supplemental Material [file KGMI_A_2316923_SM4468.zip › Figure S6.tif]

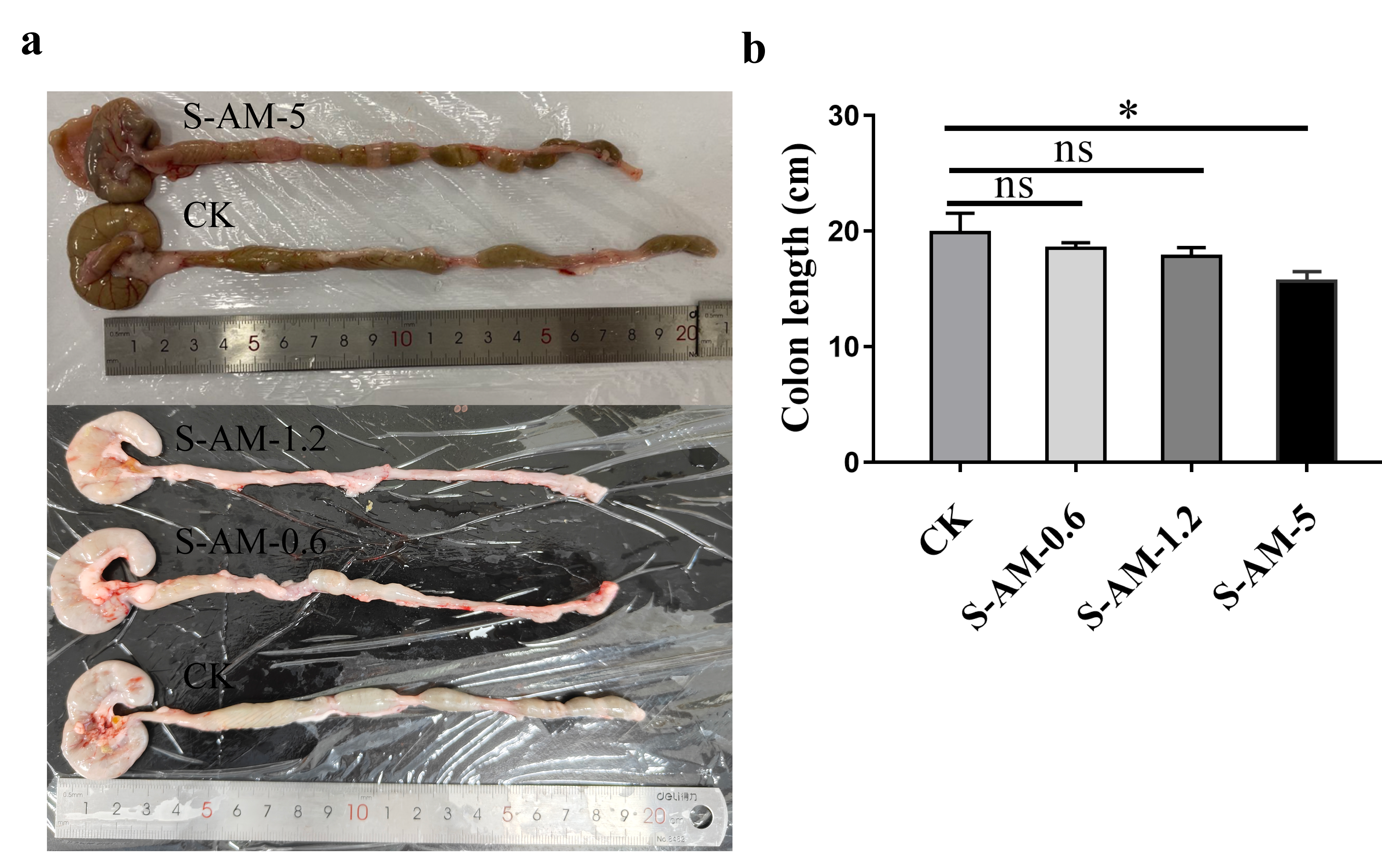

Supplement: Supplemental Material [file KGMI_A_2316923_SM4468.zip › Figure S7.tif]

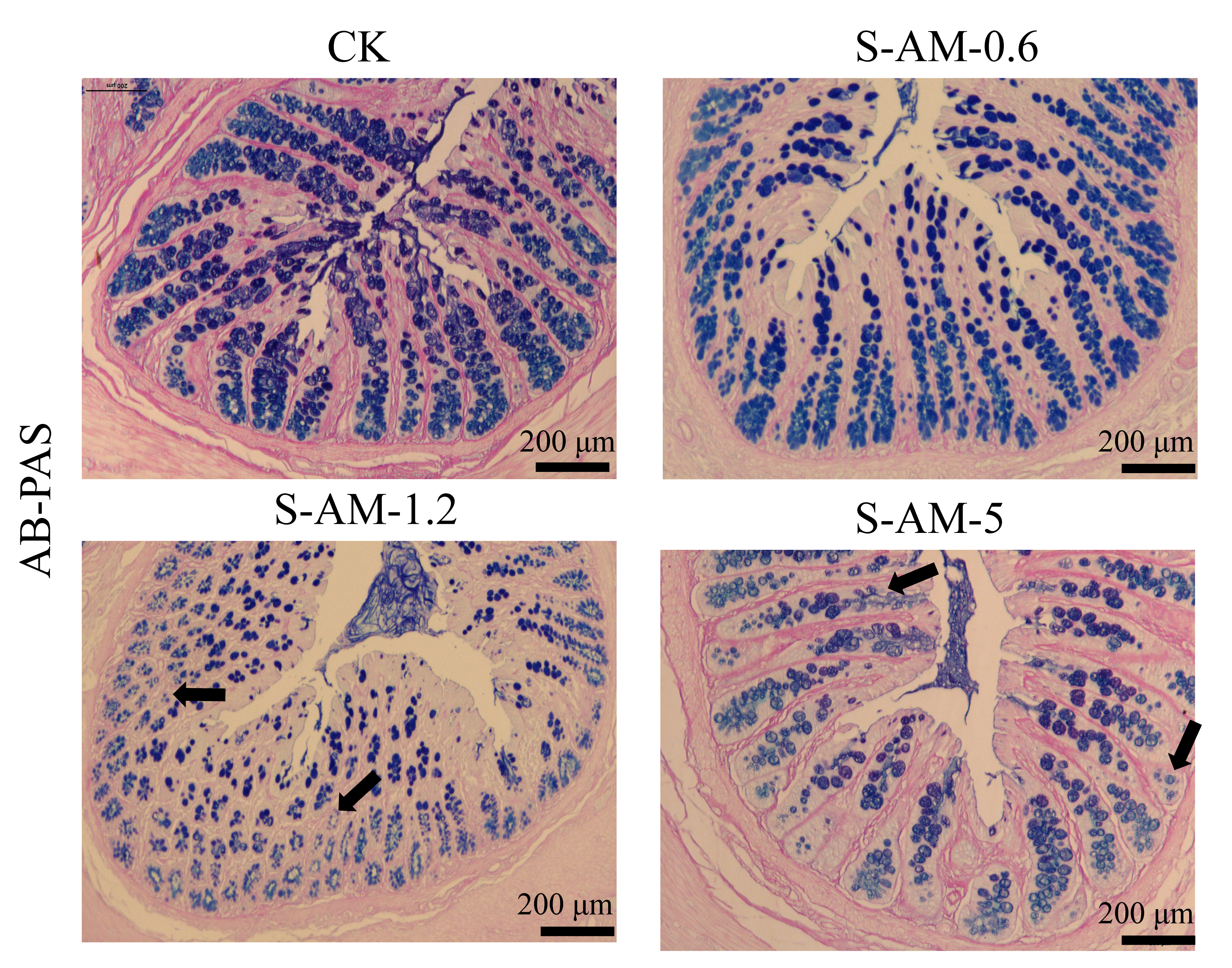

Supplement: Supplemental Material [file KGMI_A_2316923_SM4468.zip › Figure S8.tif]

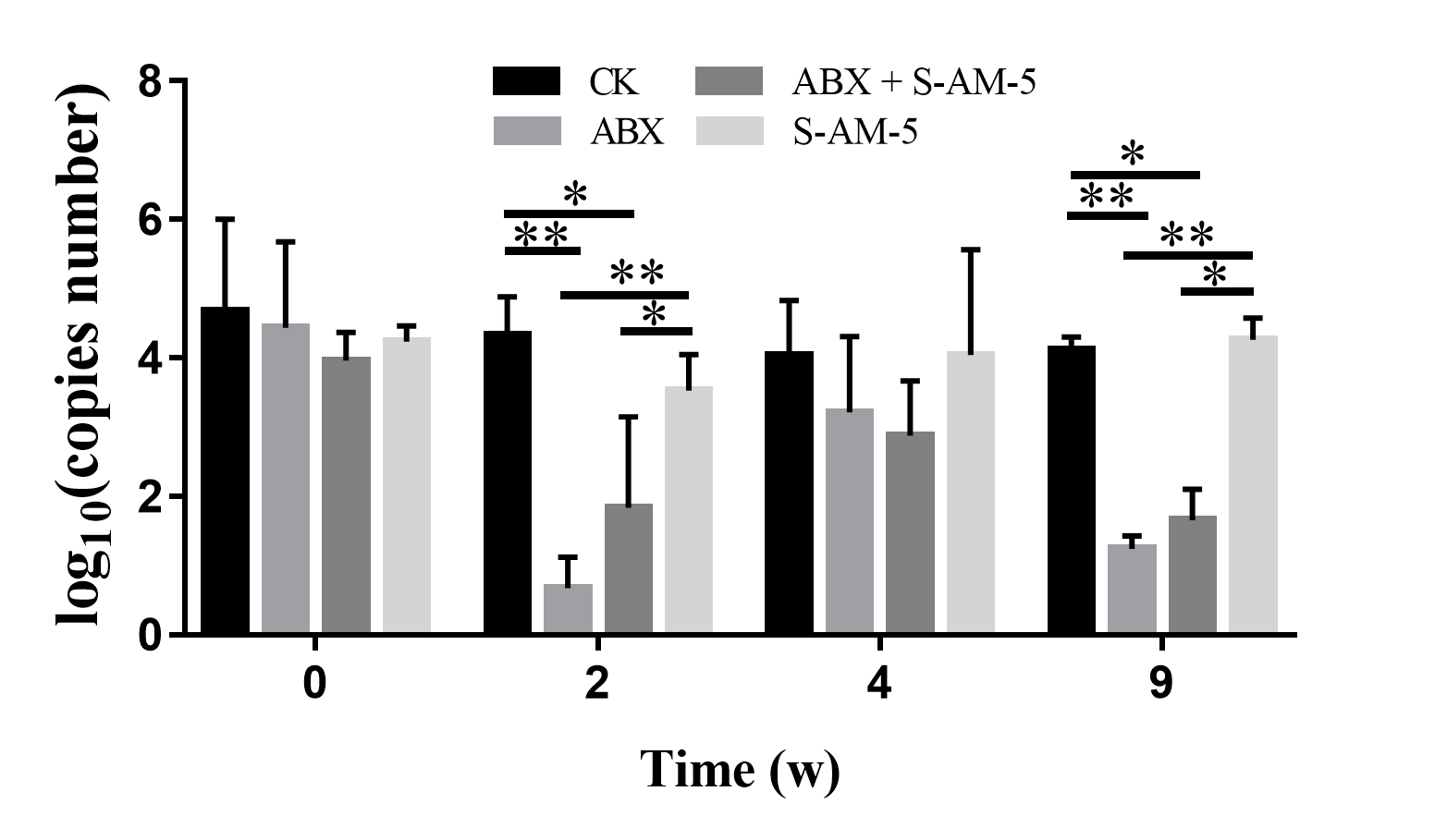

Supplement: Supplemental Material [file KGMI_A_2316923_SM4468.zip › Figure S9.tif]
