# Supplementary material for: S-amlodipine induces liver inflammation and dysfunction through the alteration of intestinal microbiome in a rat model
Source: Gut Microbes. 2024 Feb 24;16(1):2316923. doi: 10.1080/19490976.2024.2316923 (PMC10896145; doi:10.1080/19490976.2024.2316923)
Supplement: Supplementary file 1 clean.docx [file KGMI_A_2316923_SM4673.docx]

# S-amlodipine induces liver inflammation and dysfunction through the alteration of intestinal microbiome in a rat model

Xinxin Liu ^a,b^, Hui Fang ^c^, Liuzhu Pan ^c^, Peng Zhang ^a,b^, Huai Lin ^b^, Huihui Gao ^c^ , Chaolin Ye ^c^ , Daqing Mao *^,c^, and Yi Luo *^,b,a^

^a^ College of Environmental Sciences and Engineering, Ministry of Education Key Laboratory of Pollution Processes and Environmental Criteria, Nankai University, Tianjin 300350, China

^b^ State Key Laboratory of Pollution Control and Resource Reuse, School of the Environment, Nanjing University, Nanjing 210046, China

^c^ School of Medicine, Nankai University, Tianjin 300071, China

*Address corresponding to Daqing Mao, E-mail address: maodq@nankai.edu.cn;

Yi Luo, E-mail address: luoy@nankai.edu.cn.

**Supplementary methods**

**Quantification of fecal total bacteria by qPCR assays**

Bacterial DNA was extracted from 200 mg of feces using a Stool Genomic DNA Extraction Kit (D2700, Solarbio) according to the manufacturer’s protocol. Quantification of total bacteria in rat feces was conducted using primers U16Sfr (5′-TCCTACGGGAGGCAGCAGT-3′) and U16Srv (5′-GGACTACCAGGGTATCTA ATCCTGTT-3′)^1^. PCR products were cloned into pClone007 Versatile Vector (TSINGKE, China). The cloned plasmids were extracted using TIANprep Mini Plasmid Kit (TIANGEN, China) and its concentration was measured using Nanophotometer N60. A 10-fold serial dilution series of the cloned plasmids, ranging from 1×10^5^ to 1×10^9^ copies/μl, which was used to construct the standard curves for U16S. The threshold cycle values were determined using qPCR equipment (LightCycler96, Roche) for the dilutions and the process of qPCR was as follows: initial denaturation for 30 s at 95 °C, followed by 40 cycles of 5 s at 95 °C and 30 s at 60 °C, melting for 10 s at 95 °C, 60 s at 65 °C, and 1 s at 97 °C, and then 37 °C for 30 s. Here, the plasmid copy number was defined as follow: number of copies = 6.02 × 10^23^ × DNA concentration /length × 650 ×10^9^, the length is the cloned DNA fragment length. The total bacterial content of the feces was obtained from the ratio of U16S to feces weight (mg) in the fecal DNA samples. Above tests were repeated for three times.

**Supplementary figures**


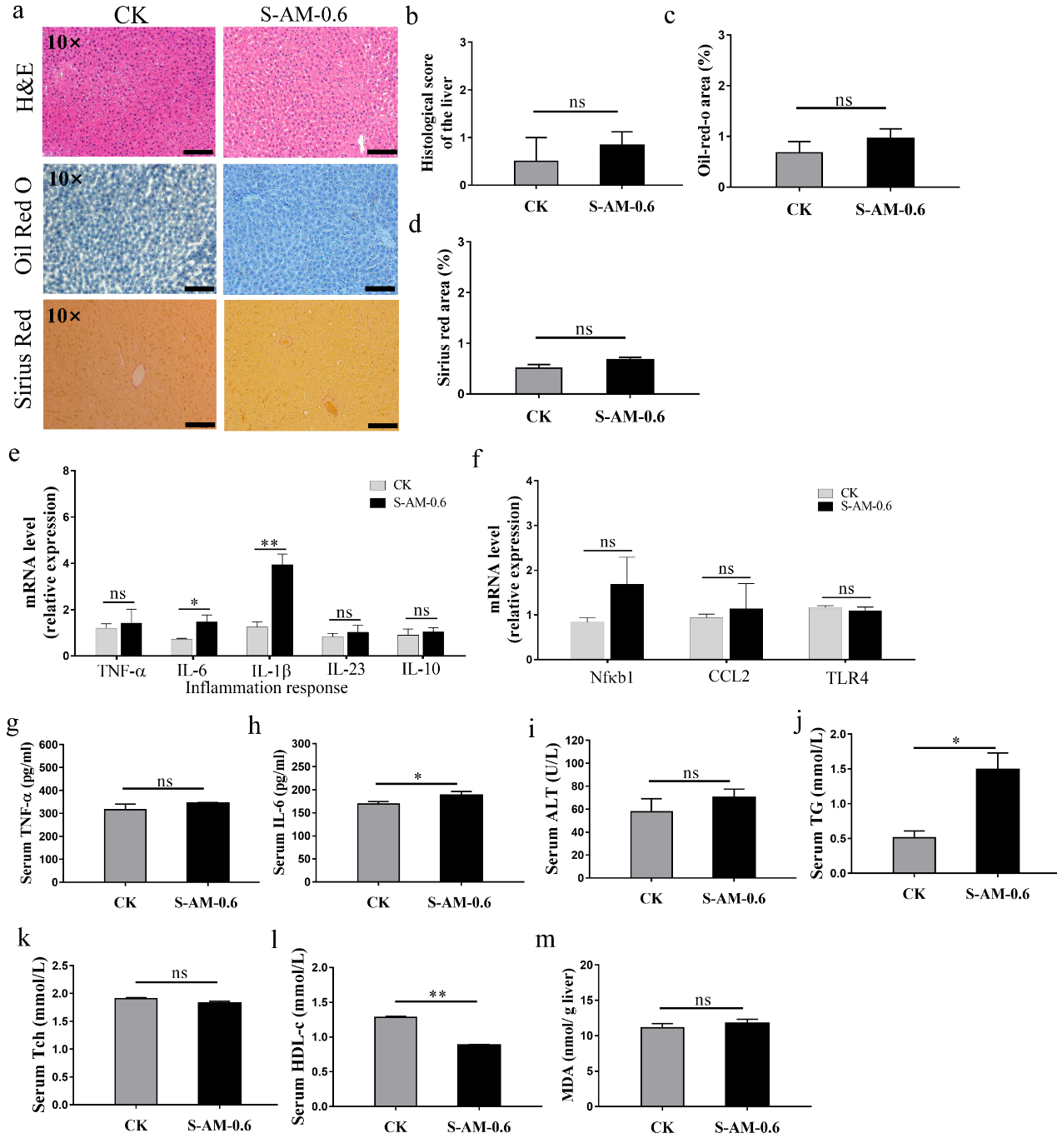


**Fig. S1.** 0.6 mg/kg of S-amlodipine did not cause hepatic inflammation and associated dysfunction in rats. (a) Representative pictures of liver sections after H&E, Oil Red O, or Sirius Red staining are shown in panels. (b-d) The histology score of the liver was evaluated (b), and the quantitation of the oil-red area (c) or the fibrosis area (d) was measured. Scale bars, 50 μm. N = 3 per group. (e) The mRNA expression of inflammatory genes in the liver were detected by RT-qPCR. N = 5 per group. (f) The mRNA expression of Nfκb1, chemokine CCL2 and TLR4 genes in the liver were detected by RT-qPCR. N = 5 per group. (g-h) Serum level of TNF-α (g), and IL-6 (h) were measured by ELISA. N = 5 per group. (i-l) The serum ALT (i), TG (j), Tch (k), and HDL-c (l) level in rats. N = 3 per group. (m) Hepatic MDA level. N = 5 per group. ALT, alanine transaminase; TG, triglycerides; Tch, total cholesterol; HDL-c, high-density lipoprotein cholesterol. MDA, malondialdehyde. Significance was determined using *t* test analysis. **P* < 0.05, ***P* < 0.01. ns, no significant difference.


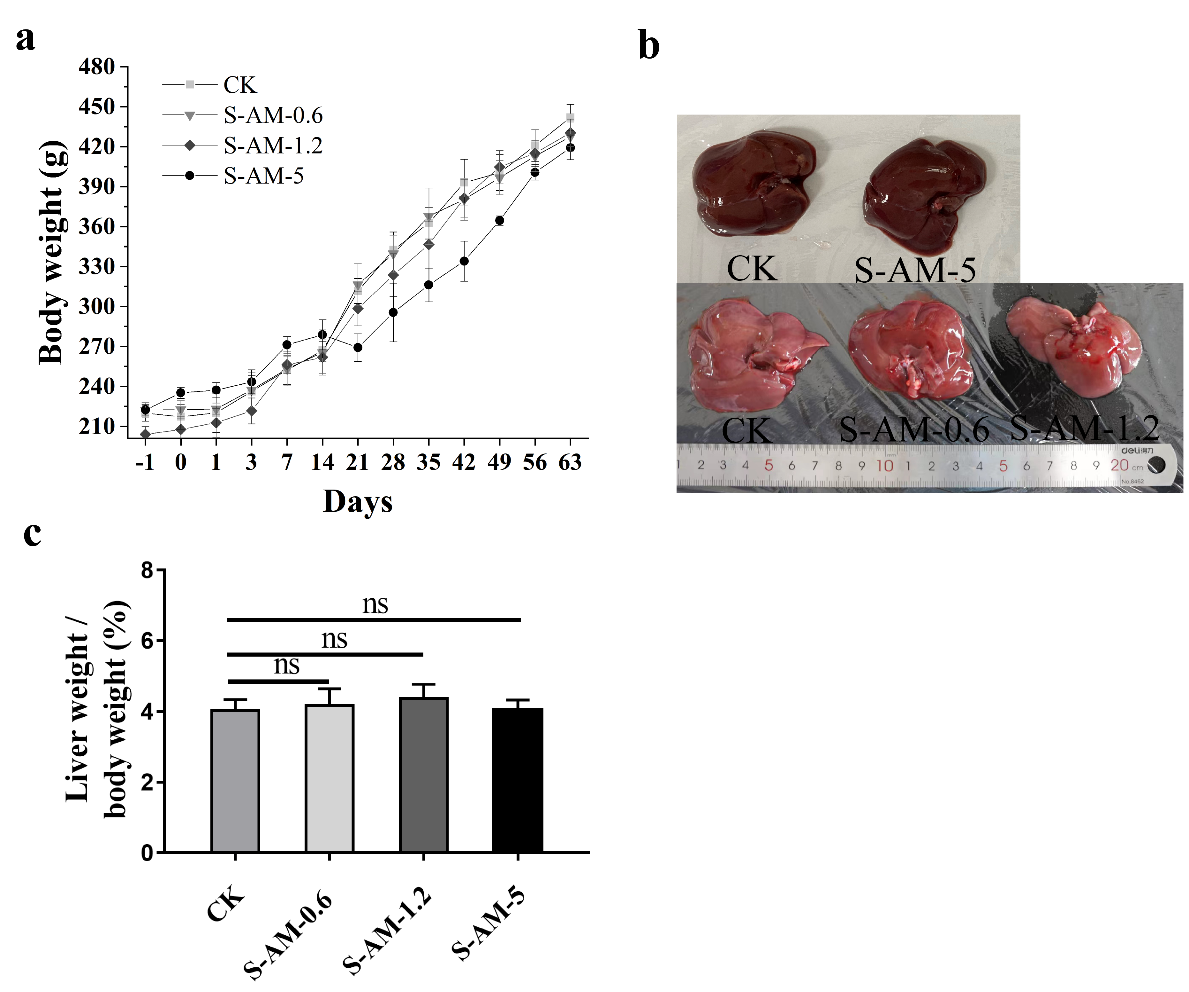


**Fig. S2.** S-amlodipine treatment resulted in a decrease in body weight without alteration the liver-to-body weight ratio. (a**)** The change in body weight over time in rats with or without S-amlodipine treatment. N = 5 per group. (b) The representative pictures of livers. N = 5 per group. (c) Ratio of liver weight to body weight. N = 5 per group. ns, no significant difference.


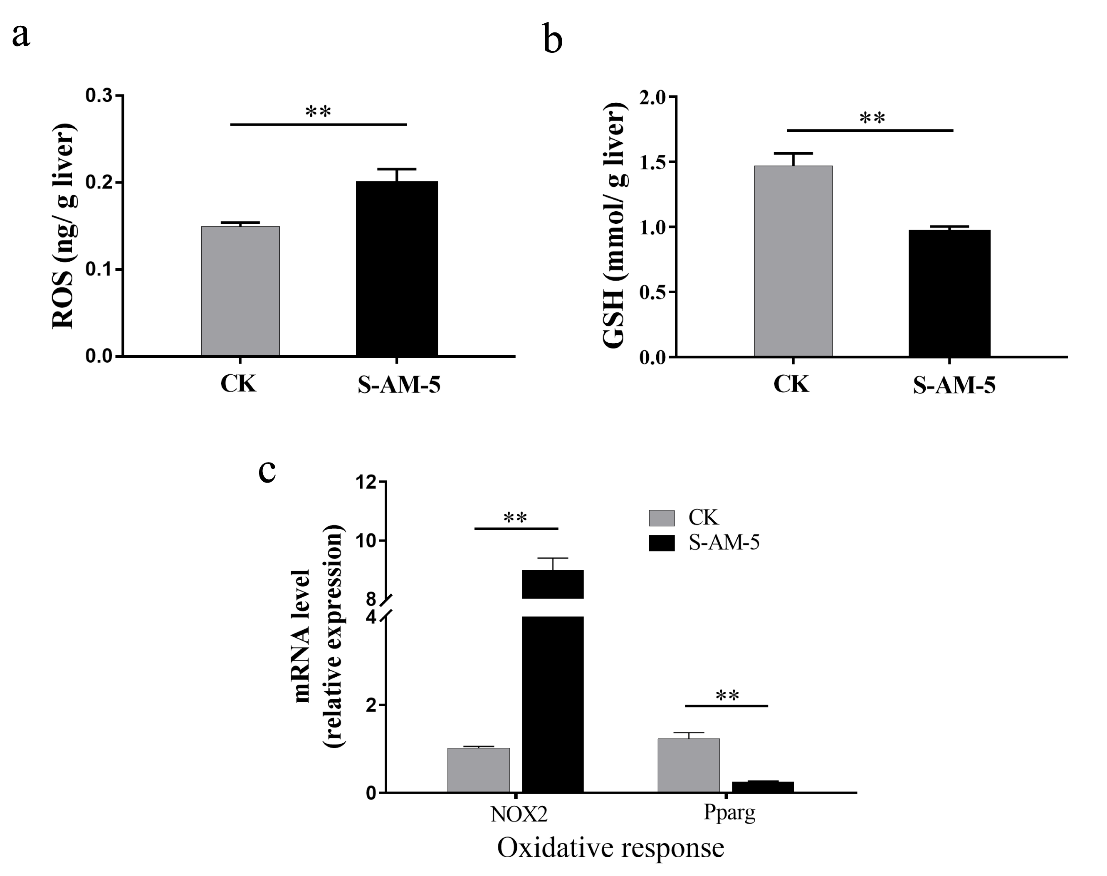


**Fig. S3.** 5 mg/kg of S-amlodipine treatment induces oxidative responses in the rat liver. (a-b) Hepatic ROS (a) and GSH (b) levels. N = 5 per group. (c) Expression of oxidative response genes (*NOX2* and *Pparg*) in the liver. N = 5 per group. **P* < 0.05, ***P* < 0.01.


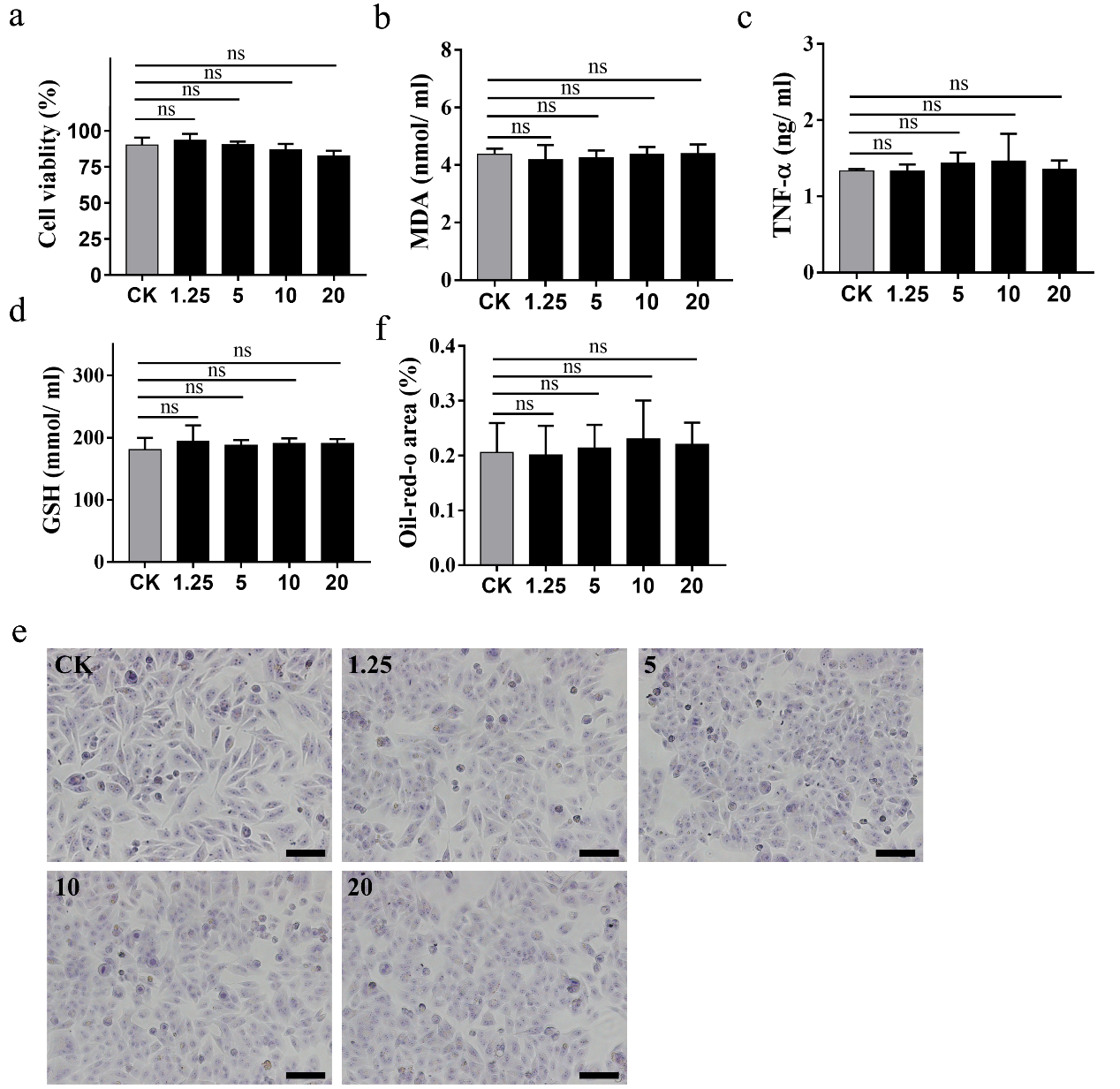


**Fig. S4.** The effects of S-amlodipine on HepG2 cells were assessed in cell experiments. (a) Cell viability was determined using the Cell Counting Kit-8 (CCK8) after continuous processing of hepatocytes with S-amlodipine (0*~20* μm) for *3 weeks*. (b-d) The levels of MDA (b), TNF-α (c), and GSH (d) were measured by ELISA in HepG2 cells after continuous treatment with S-amlodipine (0*~20* μm) for *3 weeks*. (e) Representative images of HepG2 cells stained with Oil Red O are shown. Scale bars, 50 μm. (f) The Oil-red-o area was quantified using ImageJ software. ns, no significant difference.


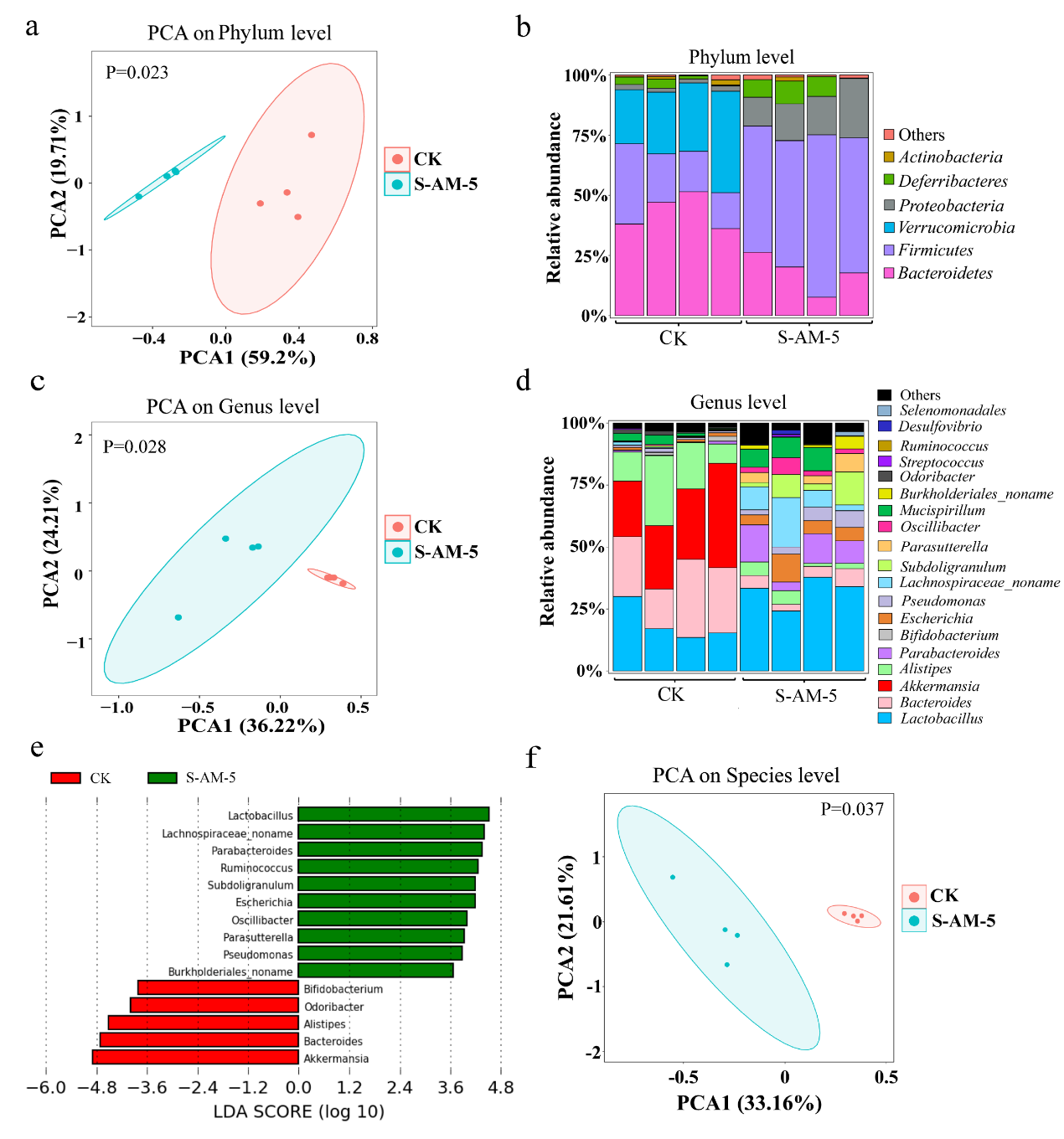


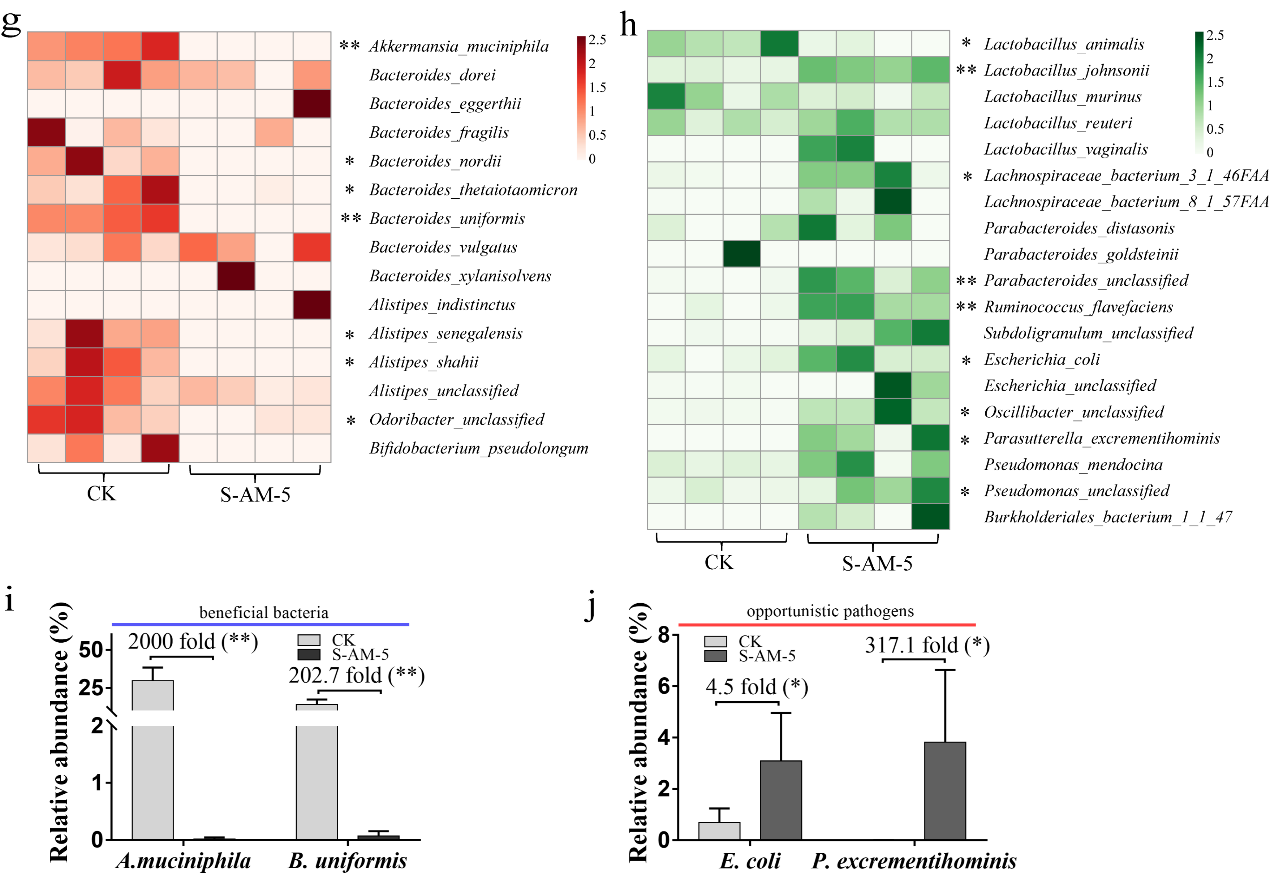


**Fig. S5.** Metagenomic sequencing analysis uncovers obvious alterations in the composition and structure of the gut microbiome following 5 mg/kg of S-amlodipine treatment. (a), (c) and (f) Principal component analysis (PCA) of eight samples (CK group (N = 4) and S-amlodipine group (N = 4)) based on phylum (a), genus (c) and species (f) level. (b) and (d) Relative abundance of fecal bacteria at the phylum level (b) and genus level (d). (e) Relative abundance of bacteria in the gut was further analyzed using LEfSe analysis. (g) and (h) Heatmaps generated using R Studio shows the taxonomic abundance at the species level based on metagenomic sequencing analysis results. The data is standardized. N = 4 per group. (i-j) Comparison of the taxonomic abundance of main species potentially associated with the production of LPS was performed based on metagenomic sequencing analysis. Significance was determined using *t* test analysis. Data are presented as the mean ± SEM. **P* < 0.05, ***P* < 0.01.


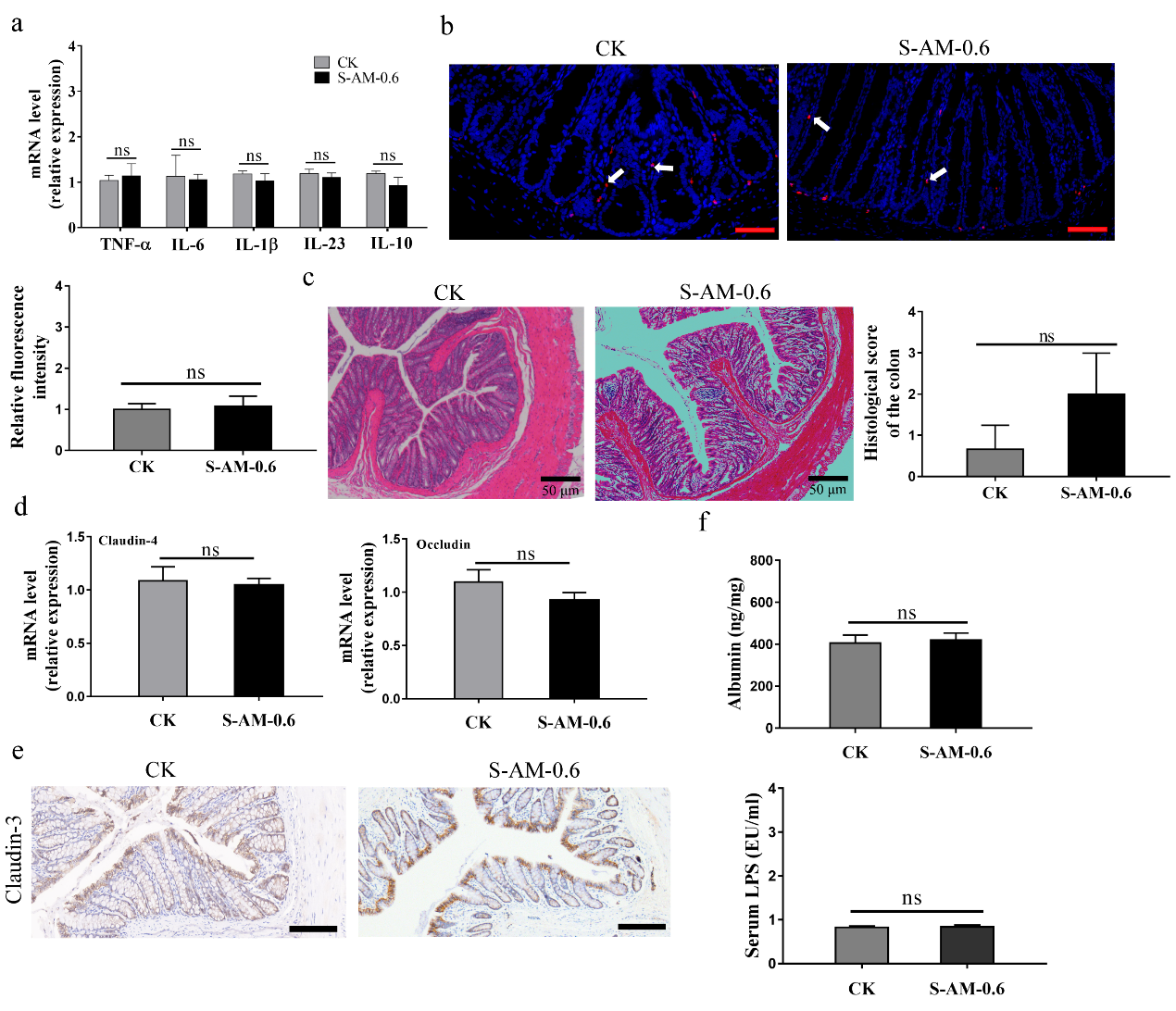


**Fig. S6.** 0.6 mg/kg of S-amlodipine did not induce intestinal inflammation and changes in colonic barrier integrity in rats. (a) The mRNA expression of inflammatory genes in the colon was detected by RT-qPCR. N = 5 per group. (b) Bacterial 16s rRNA was stained using the EUB338 probe in colon sections and the relative fluorescence intensity (FI) was calculated utilizing ImageJ software. N = 3 per group. Scale bars, 25 μm. (c) Representative images of HE staining of colons were obtained, and the histology score of the colons was determined. N = 3 per group. (d) RT-qPCR analysis on colon extracts was performed to assess the mRNA expression of Claudin-4 and Occludin. N = 5 per group. (e) Representative images of immunochemistry staining for distribution of Claudin-3 in colon tissues. N = 3 per group. Scale bars, 50 μm. (f) The fecal albumin contents and the serum LPS level in rats were measured. N = 5 per group. **P* < 0.05, ***P* < 0.01. ns, no significant difference.


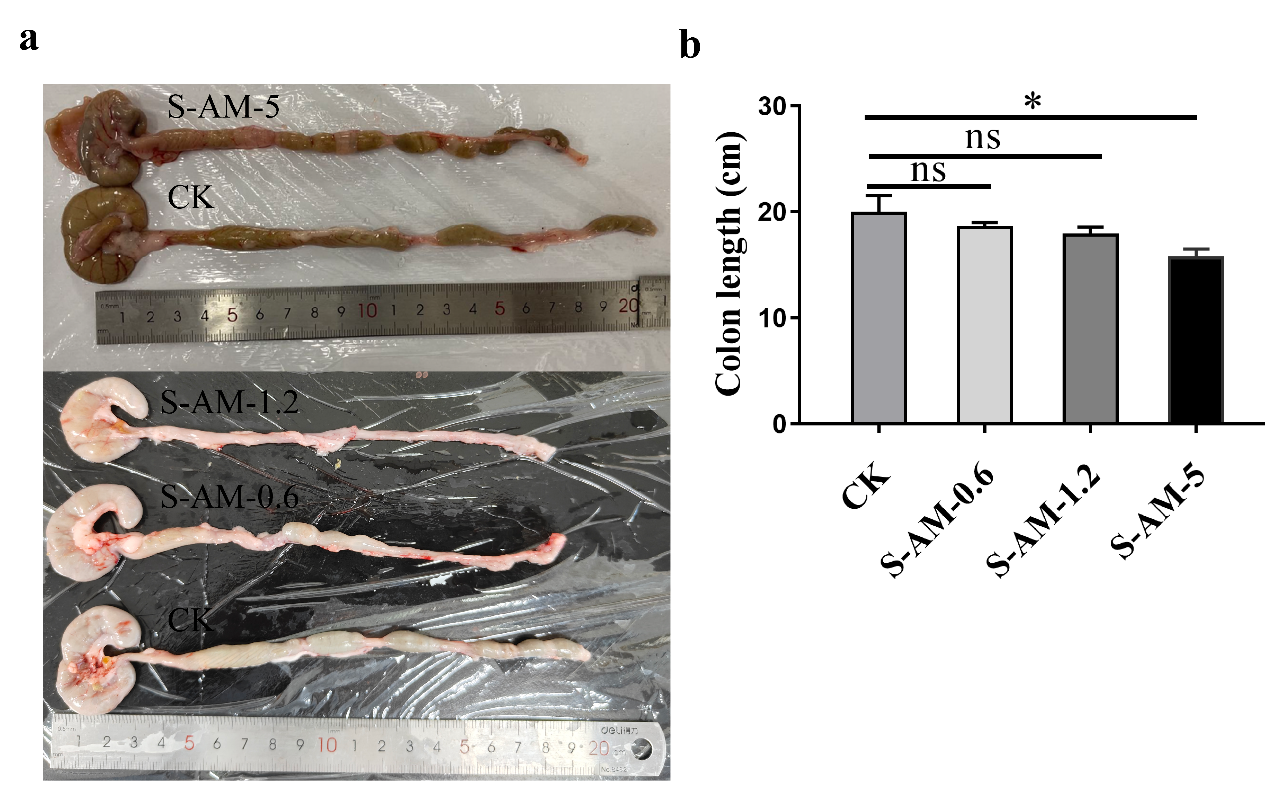


**Fig. S7.** (a) Representative pictures of colons. (b) Average colon length of rats. N = 5 per group. **P* < 0.05, ***P* < 0.01. ns, no significant difference.


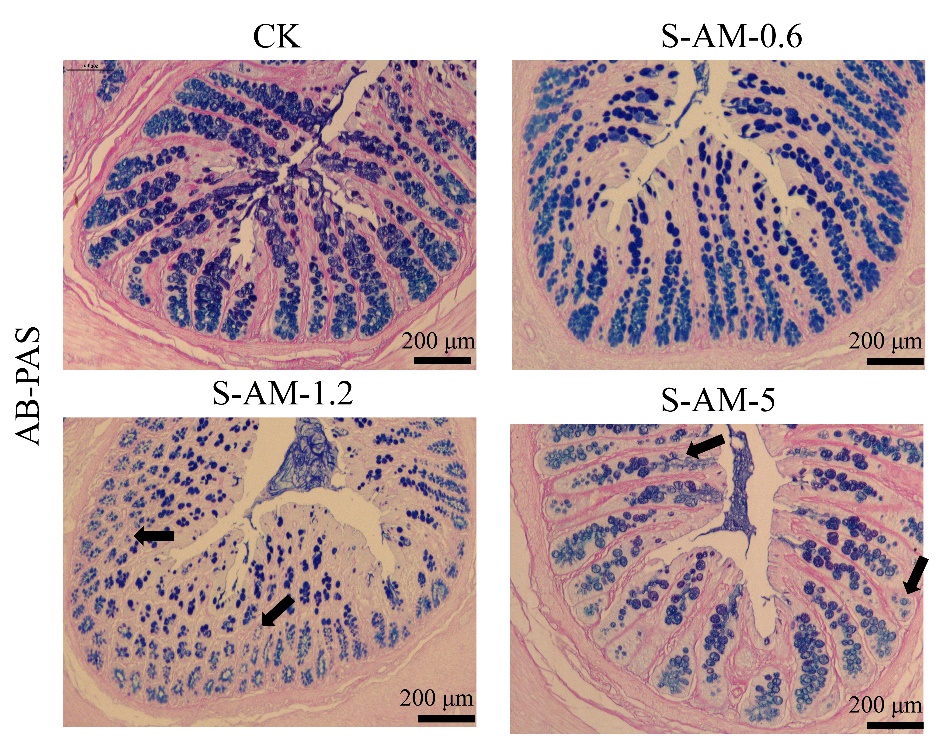


**Fig. S8.** The distribution of goblet cells in colon tissues was assessed using AB-PAS staining. N = 3 per group. Arrows indicate regions of goblet cell depletion.


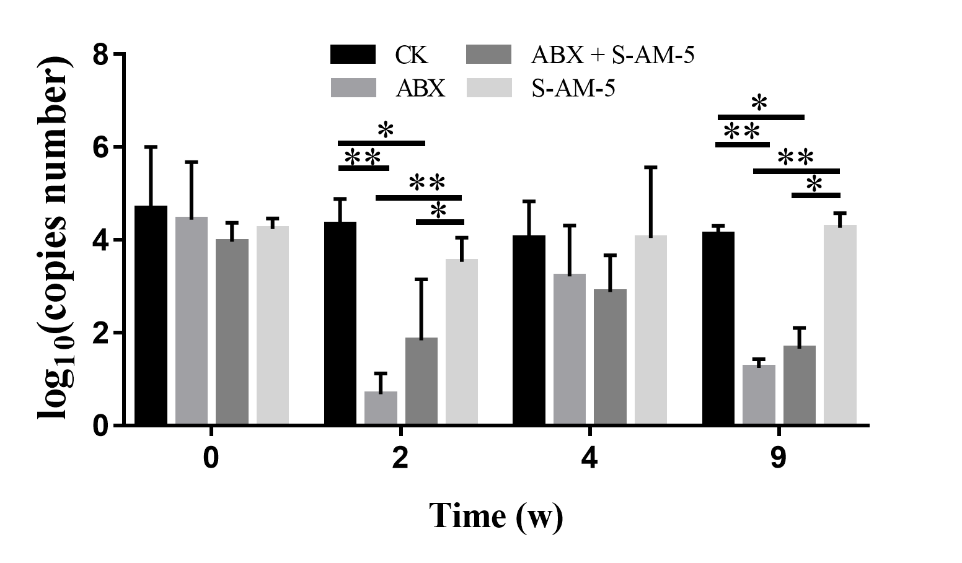


**Fig. S9.** The administration of antibiotic cocktails resulted in a reduction of the microbial load in fecal samples after 9 weeks. N = 5 per group. **P* < 0.05, ***P* < 0.01.


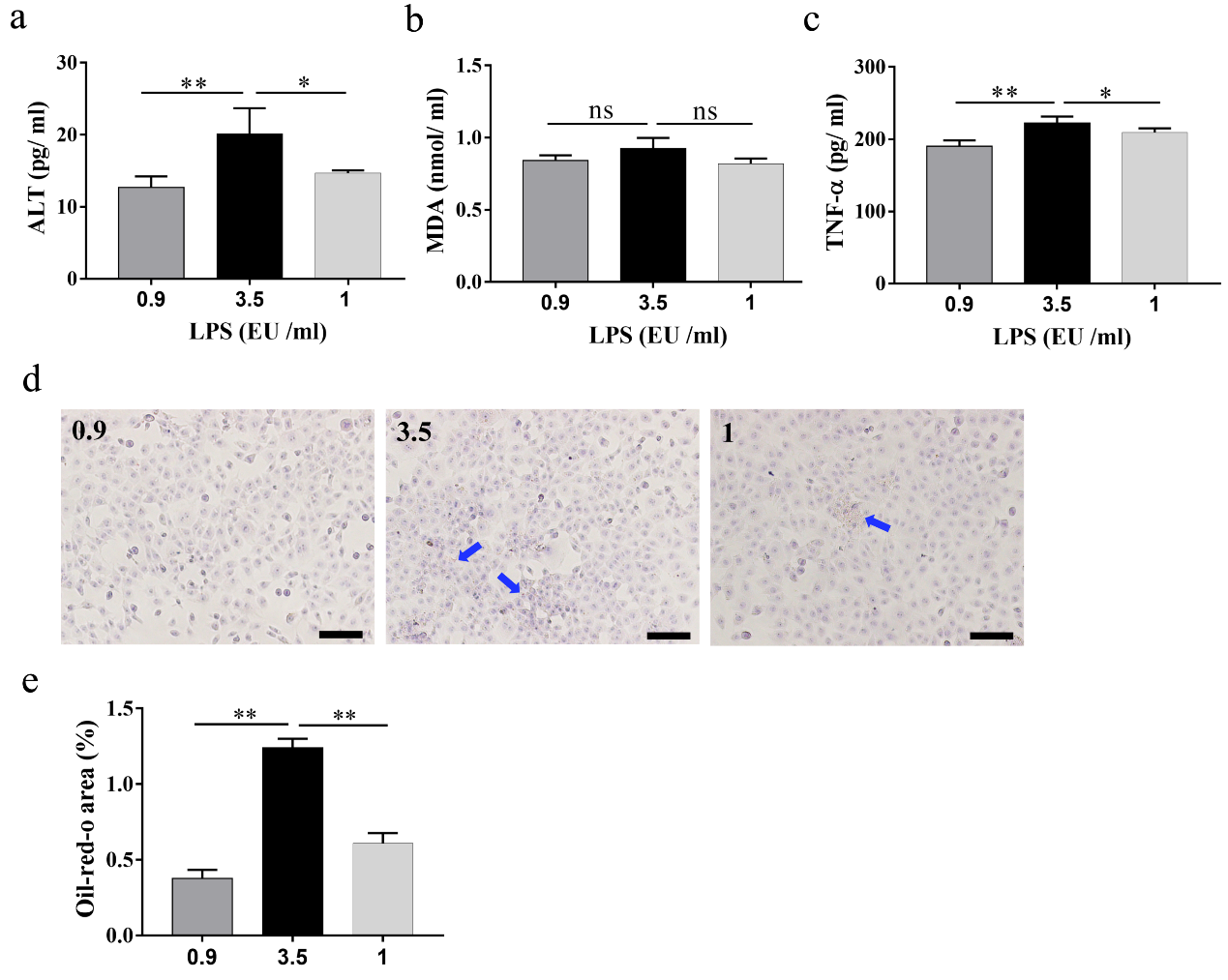


**Fig. S10.** Effects of LPS on BRL cells in cell experiments were examined. (a-c) The levels of ALT (a), MDA (b), and TNF-α (c) were measured by ELISA in BRL cells after pretreatment with LPS (*0.9*-3.5 EU/ml) for 24 h. (d) Representative images of BRL cells were obtained by Oil Red O staining after 24-hour pretreatment with LPS (*0.9*-3.5 EU/ml). Scale bars, 50 μm. Arrows indicate regions of lesions. (e) The Oil-red-o-stained area was quantified by using ImageJ software. **P* < 0.05, ***P* < 0.01. ns, no significant difference.

**Supplementary tables**

Table S1. Primers used in this study.

| Name | forward primer (5’→3’) | reverse primer (5’→3’) |
| --- | --- | --- |
| GAPDH | GGCATTGCTCTCAATGACAA | TGTGAGGGAGATGCTCAGTG |
| TNF-α | TCAGCCTCTTCTCATTCCTGC | TTGGTGGTTTGCTACGACGTG |
| IL-1β | TCGACAGTGAGGAGAATGACC | GAAGGTGCTTGGGTCCTCAT |
| IL-6 | AGTTGCCTTCTTGGGACTGA | CCTCCGACTTGTGAAGTGGT |
| NOX2 | TCAAGTGTCCCCAGGTATCC | CTTCACTGGCTGTACCAAAGG |
| HO-1 | GTAAATGCAGTGTTGGCCCC | ATGTGCCAGGCATCTCCTTC |
| Pparg | GGACGCTGAAGAAGAGACCTG | CCGGGTCCTGTCTGAGTATG |
| TLR4 | GGGGGGTATTTGACACACTCTA | TCCTTTGGATGTCTCTATGCGA |
| CCl2 | CAAAGGTGCTGAAGTCC | CAGGTGTCCCAAAGAAG |
| Tjp1 | CTGTCATGGGAGCGAAC | GAAAGGTCTAAAGGCAAGT |
| Occludin | CTGTCTATGCTCGTCATCG | CATTCCCGATCTAATGACGC |
| Claudin-4 | GGTGAAGCCCTCTACATAGG | CGTGGAAACTCCTCTGAGTG |
| Tlr9 | TGGAGTTCAGCGAGTGG | GTGCTTGATGTGGGTGG |
| Nfκb1 | TCCAGTCTCCGAGTGAAGC | GCATTCTGACCTTGCCTATC |
| IL-10 | GCAACCCAAGTAACCCT | GTCAGCCAGACCCACAT |
| IL-23 | TGCTCCGTGGGCAAAGAC | CACCACTGGGAGACTCAACA |

1. van Tilburg Bernardes E, Pettersen VK, Gutierrez MW, Laforest-Lapointe I, Jendzjowsky NG, Cavin JB, Vicentini FA, Keenan CM, Ramay HR, Samara J, et al. Intestinal fungi are causally implicated in microbiome assembly and immune development in mice. Nat Commun. 2020; 11:2577. doi:10.1038/s41467-020-16431-1.
